# Nonlinear response mechanisms of microbial communities to coral reef biogeomorphy in the South China Sea

**DOI:** 10.1128/aem.01169-26

**Published:** 2026-08-03

**Authors:** Shijie Long, Zesheng Yang, Cong Zeng, Hongqiang Yang, Ling Cao, Dazhi Wang, Yali Liu, Qiang Lin, Yue Zheng

**Affiliations:** 1 State Key Laboratory of Marine Environmental Science, College of the Environment and Ecology, Xiamen University 598522, Xiamen, China; 2 School of Oceanography, Shanghai Jiao Tong University 722366, Shanghai, China; 3 State Key Laboratory of Tropical Oceanography, South China Sea Institute of Oceanology, Chinese Academy of Sciences 74718, Guangzhou, China; 4 Nansha Marine Ecological and Environmental Research Station, Chinese Academy of Sciences, Sansha, China; 5 State Key Laboratory of Marine Environmental Science, College of Ocean and Earth Sciences, Xiamen University 534813, Xiamen, China; 6 Southern Marine Science and Engineering Guangdong Laboratory (Guangzhou) 606379 , Guangzhou, China; 7 CAS Key Laboratory of Tropical Marine Bio-Resources and Ecology, South China Sea Institute of Oceanology, Guangdong Provincial Key Laboratory of Applied Marine Biology, Chinese Academy of Sciences 74718, Guangzhou, China; 8 Marine Biodiversity and Ecological Evolution Research Center, South China Sea Institute of Oceanology, Chinese Academy of Sciences 74718, Guangzhou, China; 9 University of Chinese Academy of Sciences 74519, Beijing, China; University of Delaware, Lewes, Delaware, USA

**Keywords:** biogeomorphology, community assembly, coral reefs, functional redundancy, microbial biomarkers, South China Sea

## Abstract

To resolve how planktonic microbial composition, assembly mechanisms, and interaction architectures vary across coral reef biogeomorphic states, we sought to identify process-relevant indicators of reef resilience. We analyzed size-fractionated microbiomes from 57 stations in the South China Sea using 16S/18S rRNA amplicon sequencing. A distance-decay model defined a near-coral reef influence zone within 588 m, and reefs were classified into Poor, Moderate, and Good states based on coral density metrics. Results showed that microbial composition diverged significantly between near-coral reefs and off-coral reef zones. Although alpha diversity remained relatively stable, characteristic coral reef amplicon sequence variants (ASVs) showed clear state-associated variation across reef biogeomorphic states. The assembly of coral reef characteristic ASVs was predominantly stochastic, yet deterministic processes strengthened under intermediate conditions, indicating a transitional restructuring phase. Network complexity displayed a hump-shaped pattern, peaking in moderate states, whereas networks in good-condition reefs were sparser but more stable due to cross-kingdom interactions and increased module hubs. Ultimately, embedding microbiome analyses within a biogeomorphic framework reveals nonlinear, state-dependent microbial reorganization missed by standard richness metrics. These responsive taxa and interaction architectures jointly provide mechanistic indicators for diagnosing coral reef health in rapidly changing tropical seas.

**IMPORTANCE:**

Microbial composition, assembly processes, and interaction architecture jointly provide process-relevant indicators of reef condition and resilience across coral-reef biogeomorphic states. Although alpha diversity remained broadly stable across space and biogeomorphic grades, substantial microbial turnover, pronounced state responsiveness of characteristic taxa, and marked shifts in network organization revealed strong state dependence in microbial reorganization. Responsive taxa and interaction-architecture metrics jointly provide mechanistic, process-relevant indicators for diagnosing reef condition and resilience in rapidly changing tropical seas.

## INTRODUCTION

Despite occupying only a tiny fraction of the global ocean area, coral reefs support a disproportionate share of marine biodiversity and ecosystem services (1, 2), including coastal protection (3), fisheries (4), and biogeochemical regulation (5). Coral reefs are living landforms, in which coral reefs biogeomorphology both reflects ecosystem state and feeds back to regulate habitat configuration (6, 7). Yet, coral reefs have experienced sustained global decline since the mid-20th century, and the intensification of marine heatwaves has pushed coral reefs into increasingly frequent and spatially extensive stress (8, 9), undermining the biogeomorphology and ecosystem processes that keep coral reefs habitable. Resolving the coupled relationships between coral reefs biogeomorphology and ecosystem processes is not only a scientific priority but also a prerequisite for forecasting coral reefs’ trajectories and informing sustainable management under rapid environmental change.

Microorganisms, as central drivers of matter cycling and energy flow in coral reef ecosystems, exhibit high sensitivity to environmental changes in coral reefs (10). Importantly, microbes are not merely indicators of ecosystem change; they can actively reinforce reef degradation through microbialization feedback that intensifies hypoxia and CO_2_ release in disturbed states (11, 12). At the same time, microbes participate directly in bio-cementation and bio-erosion, contributing directly to the construction and dissolution of the coral reefs’ framework (13). Microbial communities, particularly the green alga Ostreobium together with other microbes, contribute substantially to skeletal decalcification and microbially driven bioerosion of coral carbonates (14), whereas reefal microbial crusts and microbial carbonates can contribute to framework strengthening and infilling in cryptic or low-light habitats (15). Together, these properties make microbial communities an informative, process-relevant lens for linking biogeomorphic structure to ecosystem functioning.

To date, research on coral reefs’ microbiomes has advanced along two main tracks. One focuses on broad-scale biogeographic patterning of reef-associated microbial assemblages across regions, islands, and habitat types (1). The other emphasizes symbiotic and host-associated microbiomes of key coral reef builders and associated taxa (16), especially the coral holobiont, to understand microbial roles in host health, acclimation, and stress responses (17). However, these approaches have rarely been integrated explicitly with the geomorphic complexity of coral reefs. Consequently, there is still a lack of understanding of how microbial community composition and assembly are structured across heterogeneous biogeomorphological settings, and how microbial interspecies networks confer resistance or recovery potential under disturbance. Filling this gap is essential for linking microbial ecology to the geomorphic characteristics of reef platforms.

This study uses coral reef systems in the South China Sea as a natural laboratory to examine microbial community composition, assembly, and resilience across complex coral reef biogeomorphology. The South China Sea, within the species-rich Indo-West Pacific, faces escalating climatic stress and local disturbances, with documented reef degradation (18, 19). Microbial patterns are examined along coupled environmental and geomorphic gradients. Specifically, community turnover is quantified in relation to geomorphic units and micro-niche differentiation; the relative contributions of deterministic filtering versus stochastic processes to community assembly are tested, and resilience-relevant attributes are assessed across contrasting coral reef settings. By explicitly embedding microbial organization within a biogeomorphological framework, this work provides a mechanistic basis for linking coral reefs’ form to ecosystem processes, thereby strengthening the scientific foundation for conservation and sustainable development strategies in rapidly changing tropical seas.

## MATERIALS AND METHODS

### Sample collection and biogeomorphic classification

To investigate the response mechanisms of microbial communities to variations in coral reef biogeomorphology, sample collection was conducted across 57 stations situated around the coral reefs and adjacent waters of the South China Sea. A total of 57 paired filter samples (0.22 μm and 3.0 μm) were obtained. The 3.0 μm filters primarily retained nano-/micro-eukaryotic plankton, microbial aggregates, and particle-attached prokaryotes, while the 0.22 μm filters retained the 0.22–3.0 μm fraction passing through the 3.0 μm filters, including picoeukaryotes and free-living prokaryotes (20). At each station, 10 L of surface seawater was collected using Niskin bottles. The seawater was initially pre-filtered through 200-mesh silk to exclude large zooplankton and suspended particles. Subsequently, size-fractionated filtration was performed sequentially using 3.0 μm pore-size polycarbonate (PC) membranes and 0.22 μm pore-size polyethersulfone (PES) membranes to collect microorganisms from different size fractions. The filters were immediately flash-frozen in liquid nitrogen on board and subsequently stored at −80°C in the laboratory until further experiments. Seawater temperature, salinity, dissolved oxygen, and other *in situ* physicochemical parameters were determined with an SBE 911plus CTD system (Sea-Bird Scientific, USA). Meanwhile, seawater samples intended for inorganic nutrient analysis were collected and preserved at −20°C, and the concentrations of phosphate (PO_4_-P), ammonium (NH_4_-N), silicate (SiO_3_-Si), nitrate (NO_3_-N), and nitrite (NO_2_-N) were measured using an AutoAnalyzer 3 continuous flow analyzer (Bran + Luebbe, Germany). To characterize reef-state-associated reorganization of the planktonic microbiome as a whole, primary analyses included both the >3.0 µm and 0.22–3.0 μm fractions unless otherwise stated; single-fraction analyses are identified in the text or figure legends.

To delineate the spatial extent of coral reef influence on microbial communities and identify associated characteristic microorganisms, this study conducted a comparative analysis between near-coral reef and off-coral reef zones. Microbial community entropy was calculated based on eigen-microstates using amplicon sequence variant (ASV) abundances relative to the distance from the coral reefs. Subsequently, a first-order autoregressive (AR1) model (21) was employed to fit the relationship between entropy and distance, thereby defining the near-coral reefs (NearCR) and off-coral reefs (OffCR) zones. To further validate this spatial threshold, segmented regression was performed based on pairwise Bray-Curtis community dissimilarity and pairwise distance difference (22), which identified a breakpoint close to the AR1-derived threshold and supported the NearCR/OffCR boundary. Based on long-term ecological monitoring data from the South China Sea, a classification framework for coral reef habitat quality was established using live coral cover (LCC) and juvenile coral density (JCD) as core metrics. This system integrates the current biological structure (represented by LCC) and the potential for geomorphic maintenance and recovery (represented by JCD) to categorize study sites into Poor, Moderate, and Good grades. Specifically, we classified samples as poor (severely degraded) if LCC ≤5% or JCD ≤2 ind./m^2^, and as good (relatively healthy) if LCC >30% or JCD >8 ind./m^2^. The medium stage is a transitional phase between the two, with LCC ranging from 5% to 30% and JCD between 2 and 8 ind./m^2^.

### DNA extraction and sequencing

Total DNA was extracted from all microbial samples using the phenol-chloroform-isoamyl alcohol method (23). Agarose gel electrophoresis and a NanoDrop spectrophotometer (Thermo Fisher Scientific, USA) were employed to examine the quality and concentration of the extracted DNA. The V3-V4 hypervariable regions of the prokaryotic 16S rRNA gene and the V4 region of the eukaryotic 18S rRNA gene were amplified with the primer sets 338F (5′-ACTCCTACGGGAGGCAGCA-3′)/806R (5′-GGACTACHVGGGTWTCTAAT-3′) (24–26) and 547F (5′-CCAGCASCYGCGGTAATTCC-3′)/952R (5′-ACTTTCGTTCTTGATYRA-3′) (27), respectively. The thermal conditions for PCR include an initial denaturation at 95°C for 5 minutes, followed by denaturation at 95°C for 30 seconds, annealing at 56°C for 30 seconds, extension at 72°C for 40 seconds, and finally extension at 72°C for 10 minutes, totaling 30 cycles. PCR products were verified via agarose gel electrophoresis and subsequently purified using the AxyPrep DNA Gel Extraction Kit (Axygen, America). Sequencing libraries were generated and assessed for quality using a Qubit fluorometer (Thermo Fisher Scientific, America) and an Agilent 2100 Bioanalyzer (Agilent Technologies, America). Finally, paired-end sequencing (2 × 250 bp) was conducted on an Illumina NovaSeq 6000 platform (Illumina, USA).

### Bioinformatics analysis

The original paired-end reads of amplicon sequencing were first used for quality control using Trimmomatic (v0.39) (28). The corresponding parameter settings are ILLUMINACLIP:TruSeq3-PE-2.fa:2:30:10, which is used for adapter trimming; LEADING:3 and TRAILING:3, which are used for trimming the low-quality bases at both ends; SLIDINGWINDOW:4:15, which is used for filtering the low-quality parts; and MINLEN:100, which is used for removing short reads after trimming. The obtained high-quality sequences were imported into QIIME 2 (v2023.5) (29), and denoising, read merging, and chimeric removal were performed using the DADA2 plugin (30) to obtain the ASV feature table. The qiime dada2 denoise-paired command runs under default conditions without the need for additional truncation to preserve the maximum sequence information. The denoising step generated the ASV feature table, representative ASV sequences, and denoising statistics. For phylogenetic analysis, representative ASV sequences were aligned using MAFFT (v7.520), and a phylogenetic tree was constructed using FastTree (31, 32). Taxonomic classification of ASVs was performed using a pre-trained Naive Bayes classifier based on the SILVA 138 database.

### Identification of coral reef characteristic microorganisms

To further elucidate the microbial community disparities between NearCR and OffCR zones and identify microorganisms with biomarker potential, Linear Discriminant Analysis Effect Size (LEfSe) was performed. Genera showing both statistical and biological significance between the two groups were identified using the Kruskal-Wallis test implemented in LEfSe (α = 0.05), with a Linear Discriminant Analysis (LDA) threshold >2. To construct a characteristic microbial data set reflecting different coral reefs biogeomorphic states, a multi-step filtering strategy was applied. First, coral-reef-associated microorganisms were screened from ASVs occurring in at least 10% of near-coral-reef sites, a threshold used to retain recurrent taxa and reduce rare or transient ASVs following previous microbiome studies (33), and were further identified by species enrichment analysis using false discovery rate (FDR) < 0.001 and log_2_FC > 2. Second, prevalent microbes appearing in more than 10% of samples within each condition group (Poor, Moderate, and Good) were identified. Finally, to mitigate potential bias caused by unequal sample sizes, a balanced data set comprising 54 samples, with 18 samples per Poor, Moderate, and Good category, was constructed by random subsampling based on the group with the smallest sample size. This balanced data set was used for downstream characteristic ASV identification, community assembly inference, and co-occurrence network analysis. Data analysis was conducted in R (v4.3.3) (34), mainly using the tidyverse package suite for data manipulation. Through the analysis of indicator species of nearby coral reef samples, a total of 1,162 common coral reef characteristic ASVs were ultimately identified (including 473 16S amplicon-derived ASVs and 689 18S amplicon-derived ASVs).

To identify the microorganisms significantly enriched in coral reef ecosystems under different biomorphic conditions, a differential abundance analysis was conducted using the DESeq2 method (35). The analysis was conducted on the previously filtered coral reef feature ASV matrix by comparing samples from the Poor, Moderate, and Good groups. The ASV count matrix was not rarefied before DESeq2 analysis (36). Instead, raw ASV counts were used as input, and size factors were estimated internally by DESeq2 for differential abundance testing. ASVs showing significant differences in abundance were selected using a threshold of |log_2_fold change| greater than 1.5 together with a FDR-corrected *P* value below 0.05. Biogeomorphy state bias was assigned for each significant ASV, and only ASVs consistently biased toward one biogeomorphy state were retained as differentially responsive ASVs. Random forest (37) analysis was performed in R using the ranger and caret packages after ASV filtering, CPM normalization, and log_10_ (CPM + 1) transformation. Three pairwise models among Good, Moderate, and Poor states were constructed with 2,000 trees, and 500 repeated subsampling runs were used to improve feature-selection stability. The top 100 ASVs were selected based on a composite score integrating selection frequency, variable importance, and mean ranking. Model performance was evaluated using repeated fivefold cross-validation and one-vs-rest ROC curves with corresponding AUC values (38), whereas transferability was further assessed using an independent cross-year reef data set. Representative sequences of the potential biomarker ASVs were aligned against the ASV representative sequences (39) from the cross-year reef data set, with matches retained using thresholds of percent identity ≥98.5% and query coverage >98%. To ensure comparability, the independent cross-year data set comprised 14 South China Sea reef samples collected in a different year and processed using the same sampling strategy, size-fractionated filtration, DNA extraction, primer sets, sequencing protocol, bioinformatics pipeline, and biogeomorphic classification criteria as the primary data set. This independently generated data set has not been published elsewhere.

### Community assembly process analysis

This research quantified the comparative impacts of deterministic processes (such as ecological selection) and stochastic processes (including dispersal and ecological drift) to elucidate the ecological mechanisms responsible for alterations in microbial community structure. We employed the Neutral Community Model (NCM) formulated by Sloan et al. (40) to assess the influence of stochastic processes on microbial distribution patterns. NCM assumes that all species in the community have the same birth, death, and migration rates, and their dynamics are mainly driven by randomness. Specifically, samples within each habitat condition group (Poor, Moderate, and Good) were treated as distinct metacommunities. The model was fitted separately to the ASV abundance tables of the total microbial community and the coral reef’s characteristic microbial community. Using non-linear least squares fitting, the model predicts the relationship between the frequency of occurrence of species and their mean relative abundance in the metacommunity under purely neutral assumptions. The coefficient of determination (*R*^2^) was used to check how well the model fit the data. A higher *R*^2^ value means that the neutral process can fully explain the observed data, suggesting that community aggregation is mostly random. On the other hand, the lower or negative *R*^2^ indicates that neutral processes alone poorly explain the observed occurrence-abundance relationship, suggesting that additional non-neutral processes may be involved. In addition, the model also estimated the migration rate parameter (Nm), which reflects the probability of species spreading from meta-communities to local communities.

This research employed a null model analysis framework based on phylogenetic data, as established and enhanced by Stegen et al. (41, 42), to further distinguish between different categories of deterministic and stochastic processes. This framework facilitates the direct quantification of ecological processes, eliminating the necessity to correlate community composition with explanatory variables. By integrating the β-Nearest Taxon Index (βNTI) and the Bray-Curtis-based Raup-Crick metric (RC_bray_), community assembly mechanisms were categorized into five primary processes: Heterogeneous Selection, Homogeneous Selection, Dispersal Limitation, Homogenizing Dispersal, and Undominated processes. The classification procedure begins with the calculation of βNTI values derived from null model testing of the β-Mean Nearest Taxon Distance (βMNTD). A value of |βNTI| > 2 indicates the dominance of deterministic selection, where βNTI > 2 signifies Heterogeneous Selection and βNTI < −2 signifies Homogeneous Selection. Conversely, when |βNTI| < 2, stochastic processes are considered dominant. In these cases, the RC_bray_ metric, obtained via null model testing of the taxonomic Bray-Curtis β-diversity, is employed for further differentiation. More specifically, RC_bray_ > 0.95 means Dispersal Limitation, RC_bray_ < −0.95 means Homogenizing Dispersal, and |RC_bray_| < 0.95 means that undominated processes, like ecological drift, are what make communities come together.

### Co-occurrence network analysis

Using the *ggClusterNet* (43) and *igraph* (44) packages in R, we made microbial co-occurrence networks for each habitat zone. We also calculated Pearson correlation coefficients between the total ASVs and the ASVs that are typical of coral reefs. For network construction, only strong and significant correlations (|*r*| > 0.8, *P* < 0.05) were kept. Gephi (v0.10.1) was used to visualize and explore the network. To evaluate the impact of biogeomorphic variations on network stability, robustness metrics were employed for quantification. We found the most important nodes in the networks by calculating the within-module connectivity (Zi) and the among-module connectivity (Pi) for each node. Based on these numbers, nodes were put into four topological roles: Module hubs (Zi >2.5, Pi <0.62), Connectors (Zi <2.5, Pi >0.62), Network hubs (Zi >2.5, Pi >0.62), and Peripherals (Zi <2.5, Pi <0.62). The three other categories, not including peripherals, were all called critical nodes. These nodes are crucial for keeping the network stable and structured.

## RESULTS

### Composition and diversity patterns of microbiomes in representative coral reefs of the South China Sea

**Fig 1:**
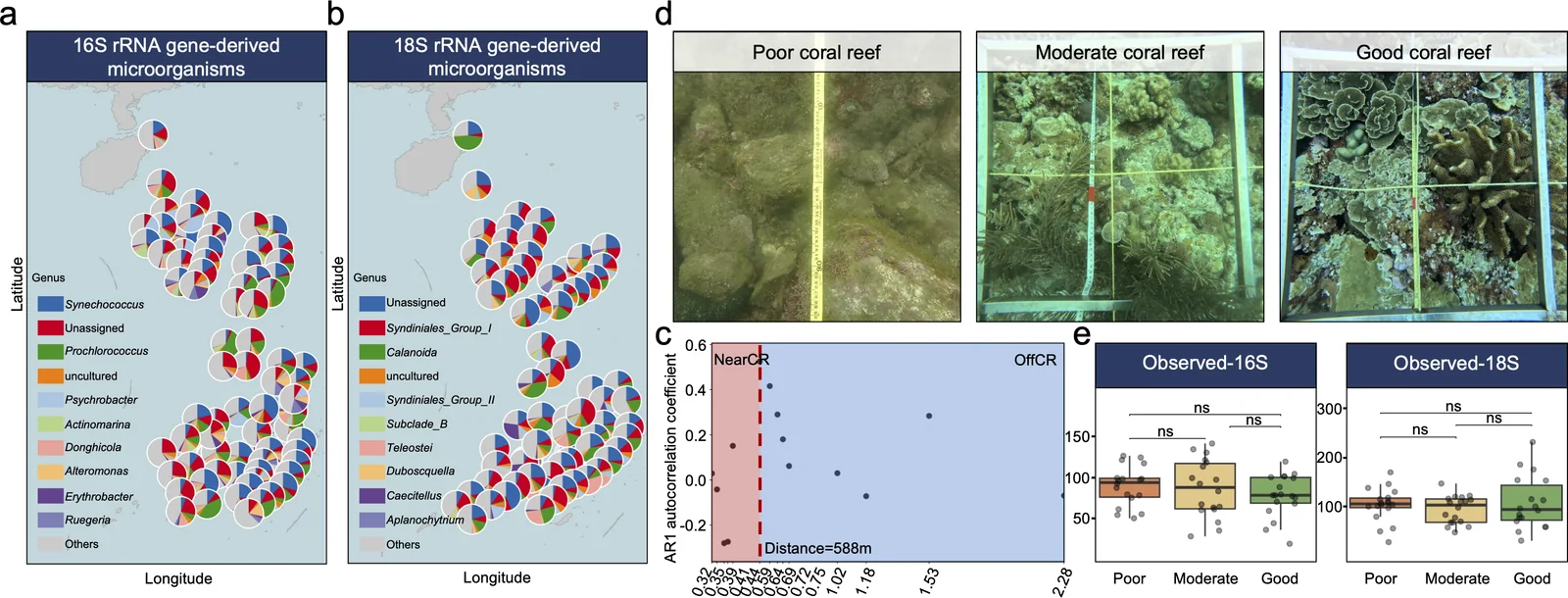
Spatial distribution patterns and diversity characteristics of coral reef microbial communities. Taxonomic composition of the top 10 most abundant 16S rRNA gene-derived microorganisms (**a**) and 18S rRNA gene-derived microorganisms (**b**) genera at each sampling site, based on 16S rRNA and 18S rRNA gene sequencing of the >3.0 µm size fraction. Each pie chart represents a sampling station, with colors corresponding to different microorganisms. (**c**) Definition of the near coral reefs boundary based on eigen-microstate entropy of the >3.0 µm fraction and the first-order autoregressive (AR1) model. The x-axis represents the distance from the coral reefs, and the y-axis represents the AR1 autocorrelation coefficient. (**d**) Classification of coral reef biogeomorphic status into three grades: Poor, Moderate, and Good. (**e**) Diversity analysis of the filtered coral reef characteristic ASVs across different biogeomorphic conditions. The alpha diversity (observed species) of 16S (left) and 18S (right) coral reefs characteristic ASVs among the Poor, Moderate, and Good groups.

Microorganisms represent one of the biotic groups most sensitive to environmental fluctuations within coral reef ecosystems. In this study, we integrated 114 microbial community samples collected from coral reefs and their surrounding waters in the South China Sea. Based on 16S and 18S rRNA gene amplicon sequencing, a total of 21,193 ASVs derived from 16S rRNA gene amplicons and 19,843 derived from 18S rRNA gene amplicons were obtained from the >3.0 µm and 0.22–3.0 μm size fractions. The 16S rRNA gene-derived microorganisms in the study area were dominated by *Synechococcus* and *Prochlorococcus* (Fig. 1a; Fig. S1a), while the 18S rRNA gene-derived microorganisms were primarily composed of *Syndiniales* and *Calanoida* (Fig. 1b; Fig. S1b). In terms of spatial distribution patterns, taxonomic composition at the family and genus levels showed sample-to-sample variation in the relative abundances of dominant taxa, with this variation being more evident at the genus level. However, no clear geographic separation in taxonomic composition was observed across samples (Fig. S2). To further quantify the spatial extent of the ecological niche influence of the coral reefs, we identified a near coral reefs zone (NearCR, within 588 m of the coral reefs center) and an off coral reefs zone (OffCR, beyond 588 m) based on community structural characteristics at the ASV level (Fig. 1c) (sample from >3.0 µm). LEfSe analysis identified 28 microbial biomarkers unique to the NearCR zone. 18S rRNA gene-derived microorganisms displayed a multi-tiered trophic structure, encompassing *Verongiida*, *Clionaida*, *Polycladida*, and *Ciliophora* (Fig. S3a). The enriched 16S rRNA gene-derived microorganisms were mainly heterotrophic bacteria, such as *Ruegeria*, *Nautella*, and *Winogradskyella* (Fig. S3b). These findings further substantiate the essential function of coral reefs in determining the structure of adjacent marine ecosystems, thereby endorsing the “Island Mass Effect” theory, which asserts that coral reefs serve as physical barriers and nutrient reservoirs that can profoundly affect the composition of nearby planktonic microbial communities (45, 46).

However, in contrast to the differentiation observed in community structure, alpha diversity across the spatial dimensions of different coral reefs exhibited high stability. Statistical tests indicated no significant differences in the Observed Species index between NearCR and OffCR zones for either 16S/18S rRNA gene-derived microorganisms (*P* > 0.05; Fig. S3c); this trend was consistent across other alpha diversity indices (Fig. S3c and S4). This suggested that the NearCR and OffCR zones maintain similar species pool capacities despite differing habitat environments. Microorganisms are proven to act as both architects of and responders to coral reef biogeomorphology (47). To investigate the association between coral reef biogeomorphology and microbial systems, we analyzed microbial communities within the NearCR zone across different biogeomorphic classifications (Fig. 1d). Rarefaction curve analysis indicated that sequencing depth was sufficient to cover the vast majority of species in all samples across different biogeomorphic conditions (Fig. S3d). On this basis, the alpha diversity of 16S rRNA gene-derived microorganisms and 18S rRNA gene-derived microorganisms showed no significant changes across the gradient of Poor, Moderate, and Good biogeomorphic conditions (*P* > 0.05; Fig. 1e). These results suggest that alpha diversity alone may be insufficient to capture microbial community responses to reef biogeomorphic states. The theory of functional redundancy (48) may explain the underlying mechanism. This theory says that when the environment changes, the number of sensitive species goes down. However, opportunistic species that are functionally similar but more adaptable quickly fill their ecological niches. This constant change between taxa keeps the total number of species stable, but it could also cause big changes in how the whole community works. Because of this, alpha diversity indices were not good enough to measure the states of coral reef biogeomorphology because they did not show how species changed in the community. So, to get an accurate picture of the health of coral reef ecosystems and how they are breaking down, we need to take a broad view that includes community structure, the dynamics of key microorganisms, and networks of species interactions.

### Community assembly mechanisms under different coral reefs biogeomorphological conditions

**Fig 2:**
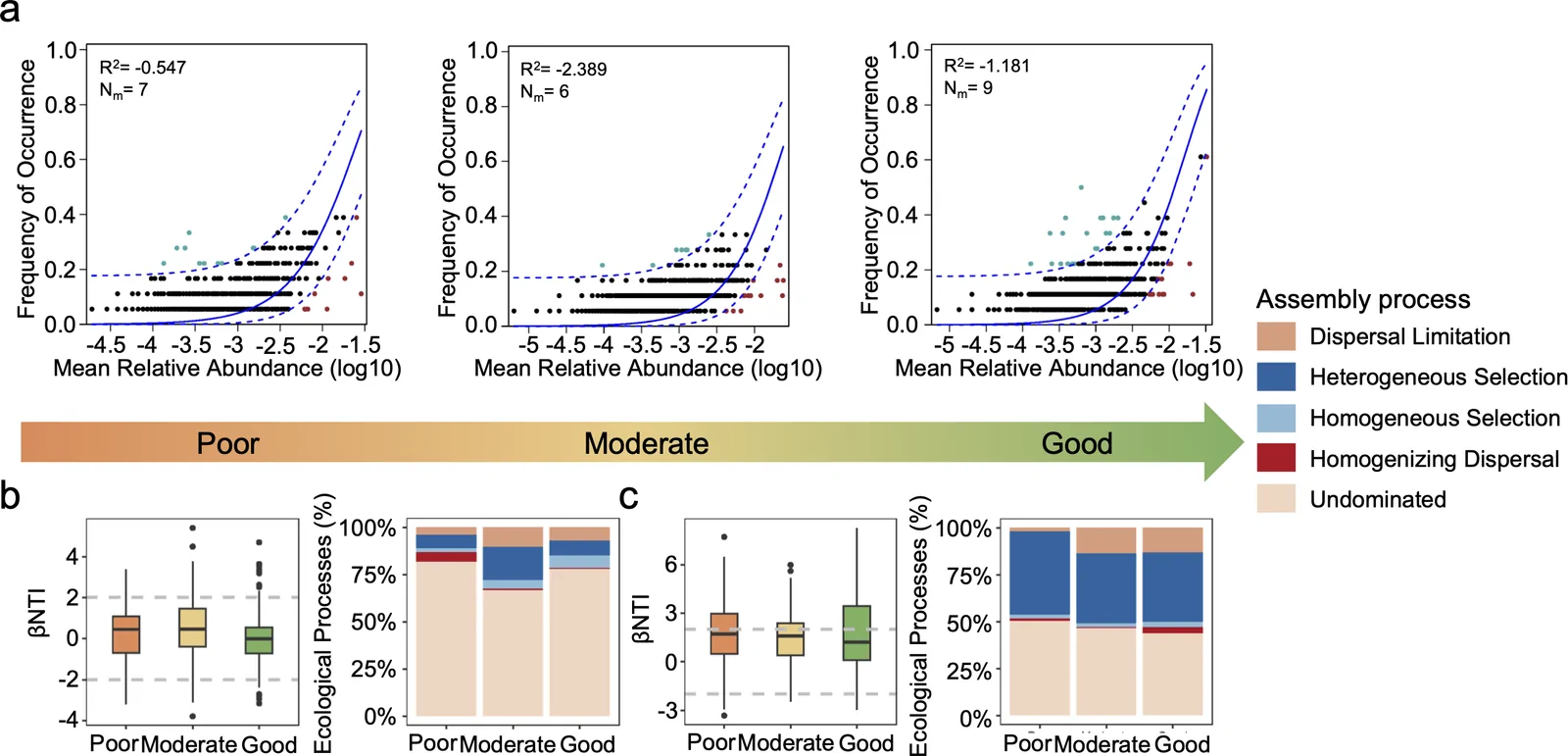
Assembly mechanisms of coral reef characteristic microbial communities. (**a**) NCM fitting results for coral reef characteristic microbial communities. This model evaluates the role of stochastic processes (dispersal and ecological drift) in community assembly. The three panels correspond to reef health conditions: Poor, Moderate, and Good. The x-axis represents the mean relative abundance of species (log_10_-transformed), and the y-axis represents the frequency of occurrence. The solid blue line indicates the model prediction, while dashed lines represent the 95% confidence intervals. Black dots represent individual ASVs. *R*^2^ indicates the goodness-of-fit, and Nm represents the estimated migration rate. (**b and c**) Phylogenetic null model analysis quantifying the contribution of different ecological processes to community assembly. Left panels: Boxplots showing the distribution of the βNTI. Pairwise comparisons with βNTI > 2 and βNTI < −2 indicate heterogeneous selection and homogeneous selection, respectively, whereas comparisons with |βNTI| < 2 were further classified using RC_bray_. Right panels: Stacked bar charts showing the relative contribution of five ecological processes: dispersal limitation, heterogeneous selection, homogeneous selection, homogenizing dispersal, and undominated stochastic processes. (**b**) Coral reef characteristic 16S rRNA gene-derived microorganisms and (**c**) coral reef characteristic 18S rRNA gene-derived microorganisms.

To elucidate the assembly mechanisms of microbial communities under varying coral reef biogeomorphic conditions, we focused on common coral reef characteristic ASVs (defined as ASVs appearing in >10% of samples across all biogeomorphic conditions) and integrated the neutral community model (NCM) with a phylogenetic null model. The analysis demonstrated that community assembly is driven by both deterministic and stochastic processes. First, the neutral community model showed a poor goodness of fit for characteristic microorganisms (*R*^2^ <0; Fig. 2a). This result indicated that the occurrence frequency-relative abundance relationships of the integrated characteristic ASV pool deviated from the expectations of the NCM. However, a low or negative NCM fit does not directly indicate deterministic dominance; rather, it suggests that neutral processes alone cannot fully explain the observed distribution patterns. The βNTI analysis was conducted to further quantify deterministic and stochastic processes. The results showed that most pairwise community turnover fell within |βNTI| < 2, indicating that strong deterministic selection was not dominant for these characteristic microorganisms (Fig. 2b and c). This trend was also confirmed by quantitative decomposition of ecological processes, which showed that stochastic processes (including dispersal limitation, homogenizing dispersal, and undominated stochastic processes) contributed 84.75% ± 6.58% and 58.17% ± 3.98% to the assembly of common 16S and 18S rRNA gene-derived characteristic microorganisms, respectively (Fig. 2b and c). However, when broadening the perspective to the entire community encompassing all species, the assembly mechanism was distinctly dominated by deterministic processes (accounting for 59.40% and 65.48% for prokaryotes and eukaryotes, respectively; Fig. S5). Collectively, these results suggested that the formation of common coral reef characteristic microorganisms was co-driven by deterministic and stochastic processes. This mechanism suggested that, despite environmental filtering, high functional redundancy and niche overlap among core microorganisms allowed ecological drift to become a dominant force in community assembly. Under different biogeomorphic conditions, the assembly of common coral reef characteristic 16S rRNA gene-derived microorganisms was dominated by stochastic processes in both the Poor and Good stable states (with contribution rates of 90.85% and 85.62%, respectively; Fig. 2b). Consistent with the theory of functional redundancy (48), these results indicated that while environmental selection drove the initial assembly, stochastic processes dominated the turnover of these taxa during stable phases. Specifically, after habitat filtering created functional groups, the local distribution of ecologically similar taxa was mostly controlled by random ecological drift (49).

In the Moderate state, on the other hand, the contribution of stochasticity fell to 77.78%, while the contribution of deterministic processes rose from 9.15% to 22.22%. This shows that this transitional period is an important time for community restructuring, when environmental selection pressure is temporarily increased. The assembly mechanism of common eukaryotic microorganisms exhibited a clear successional pattern along the health gradient (Fig. 2c). Under Poor biogeomorphic conditions, deterministic (46.41%) and stochastic (53.59%) processes were co-dominant. As health conditions improved, the contribution of deterministic processes gradually decreased to 39.87%, while the cumulative contribution of stochastic processes rose to 60.13%. This meant that the pressure of environmental selection on 18S rRNA gene-derived characteristic microorganisms gradually decreased as the ecosystem recovered, which means that stochastic processes play a bigger role. In summary, the assembly of common 16S rRNA gene-derived microorganisms exhibited high stochasticity at both extremes of habitat quality (Poor and Good) but enhanced determinism in the intermediate state, reflecting unique selection pressures in transitional habitats. In contrast, common 18S rRNA gene-derived microorganisms exhibited a direct response to environmental stress. As biogeomorphic conditions degraded, the relative contribution of deterministic processes increased, suggesting that harsh habitats imposed stricter environmental filtering on 18S rRNA gene-derived microorganisms.

### Key microorganisms responsive to changes in coral reef biogeomorphology

**Fig 3:**
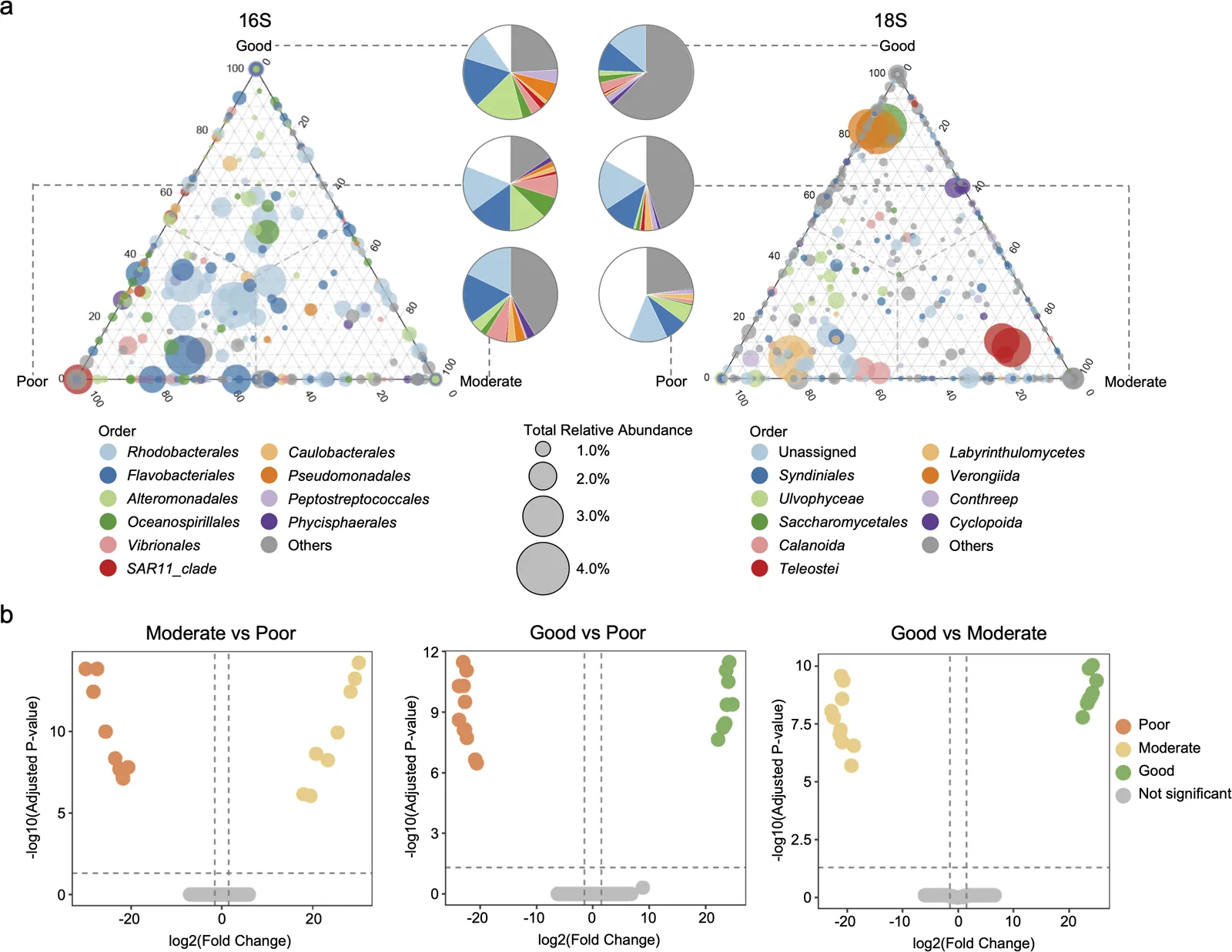
Niche preference and differential enrichment analysis of characteristic microorganisms of coral reef. (**a**) Ternary plots of niche preference and taxonomic composition. The left panel represents 16S rRNA gene-derived microorganisms, and the right panel represents 18S rRNA gene-derived microorganisms. In the ternary plots, each dot represents an individual ASV, with its position reflecting the ASV’s preference based on its mean relative abundance across Poor, Moderate, and Good biogeomorphic conditions. The size of each dot is proportional to the total relative abundance, and colors correspond to taxonomic orders. The adjacent pie charts quantify the proportional composition of species at the order level within each dominance category (i.e., sets of ASVs preferentially enriched in Poor, Moderate, or Good conditions). (**b**) Volcano plots of differential abundance analysis. These plots display ASVs showing significant abundance changes between different biogeomorphic status groups (Moderate vs Poor, Good vs Poor, and Good vs Moderate). The x-axis represents the log_2_ fold change, and the y-axis represents the −log_10_ adjusted *P*-value. Dashed lines indicate the filtering thresholds of FDR < 0.05 and |log_2_ Fold Change| > 1.5. Orange, yellow, and green points indicate ASVs significantly enriched in the Poor, Moderate, and Good conditions, respectively.

To systematically analyze the response patterns of key microorganisms to changes in coral reef biogeomorphology, we conducted a comprehensive analysis of responsive microorganisms. First, we used a ternary plot analysis (Fig. 3a) to identify which niches different biogeomorphic conditions preferred. Three main orders (*Rhodobacterales*, *Flavobacteriales*, and *Alteromonadales*) always made up the majority of 16S rRNA gene-derived communities in all states. However, the relative proportions of these orders changed a lot depending on habitat quality. The Poor biogeomorphic state had a community structure that was mostly made up of *Rhodobacterales* (19.9% of the total), followed by *Flavobacteriales* (18.4%) and *Alteromonadales* (15.6%). This predominance of *Rhodobacterales* may be ascribed to their capacity to exploit substantial quantities of organic detritus released after coral mortality or bleaching (50). In the Good biogeomorphic state, the community structure changed, and the numbers of *Flavobacteriales* (19.1%) and *Alteromonadales* (18.5%) were higher than the numbers of *Rhodobacterales* (11.5%). This change in dominant microorganisms was probably due to healthy coral reefs having unique substrate types that favor certain metabolic groups, like complex polysaccharides in coral mucus (16, 51). 18S rRNA gene-derived microorganisms exhibited substantial variations in group characteristics. *Syndiniales* (13.7%) and *Ulvophyceae* (11.2%) were the most common types of plants in the Poor state. The Good state had much higher *Calanoida* (3.5%) and *Saccharomycetales* (2.4%) than the other states. These taxa are important links between the microbial loop and higher trophic levels (52). This enrichment showed that the structure of the food web was more complicated. The results showed a strong link between how microbes respond to biogeomorphic changes. 16S rRNA gene-derived microorganisms show how environmental conditions change by changing their numbers, and eukaryotes show how ecosystems change by changing their taxonomic distribution.

Furthermore, random forest analysis and differential enrichment analysis were used to identify potential biomarker ASVs associated with coral reef biogeomorphy states (Fig. 3b; Fig. S6). Differential enrichment analysis identified 32 ASVs, whereas the random forest model identified 100 ASVs. After removing overlapping ASVs, a total of 104 unique ASVs were retained for further evaluation as potential biomarkers of reef biogeomorphy status (Table S1). To further validate the effectiveness of these 104 potential biomarker ASVs in reflecting reef biogeomorphic status, we applied them to an independent South China Sea reef data set collected in another year. These potential biomarker ASVs correctly predicted reef biogeomorphy status for 11 of 14 samples, corresponding to an accuracy of approximately 78% (Table S2). Together, these results support the effectiveness of combining random forest modeling with differential enrichment analysis in identifying ASVs with potential as biomarkers of reef biogeomorphic status.

### Microbial co-occurrence networks across coral reefs biogeomorphological states

To reveal the response mechanisms of microbial interspecies interactions to changes in coral reef biogeomorphology, co-occurrence networks of coral reef characteristic microorganisms were constructed to systematically analyze potential interactions from the perspectives of overall topology, sub-network dynamics, stability, and key nodes. The topological complexity of co-occurrence networks exhibited a hump-shaped trend (increasing then decreasing) in response to habitat quality, a pattern consistent with the intermediate disturbance hypothesis (53) (Fig. S7, Table S3). The Moderate state had the most edges (10,095) and the highest average degree (27.28), but the Good state had the fewest edges (7,400) and the lowest average degree (20.39). This change in structure was followed by a steady drop in network density (from 0.04 to 0.03), which shows that microbial networks in the Good state are likely to be less dense and more streamlined. This could be a sign of optimizing ecosystem functions.

**Fig 4:**
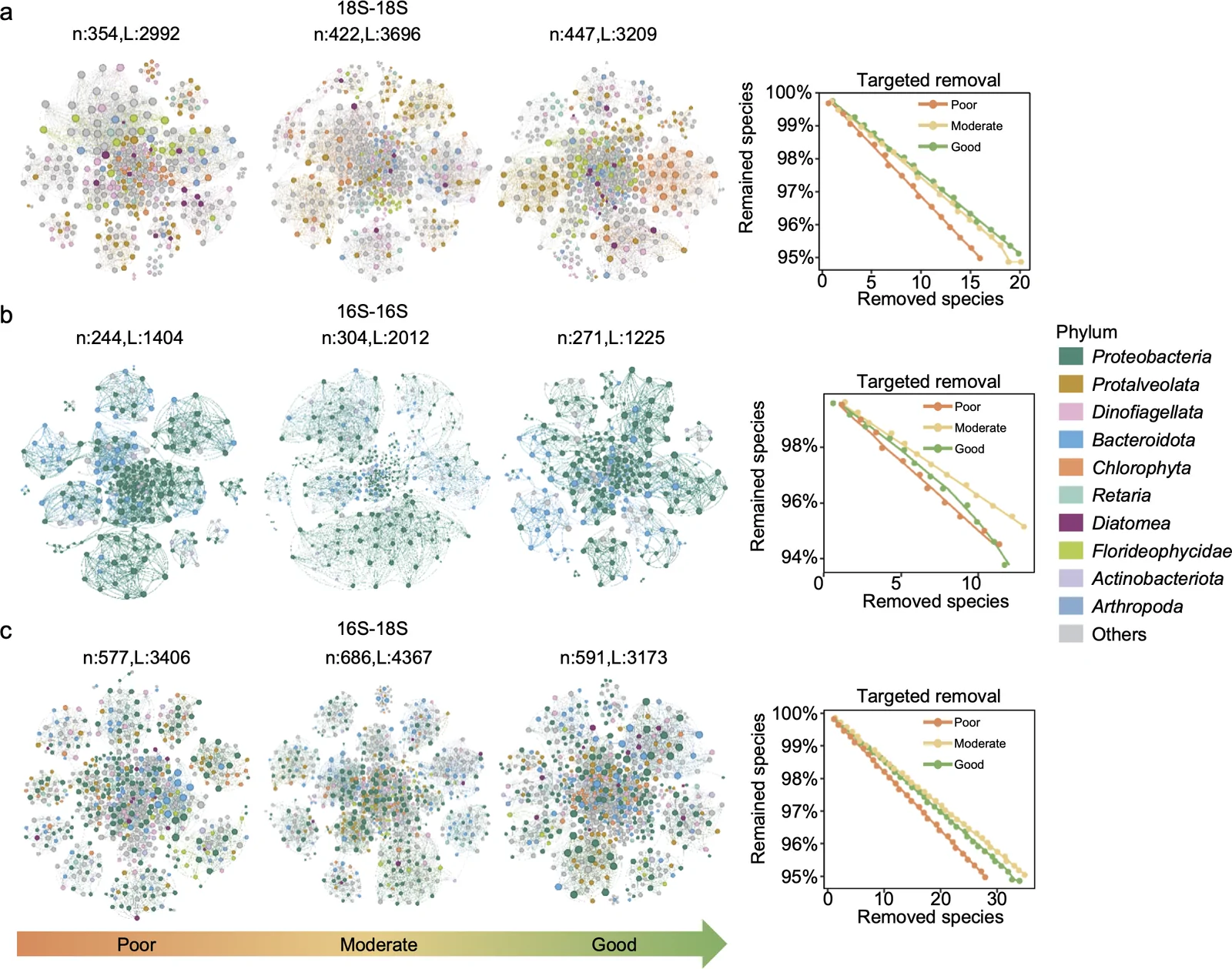
Microbial co-occurrence networks and stability analysis based on coral reef characteristic ASVs. In the co-occurrence network, nodes represent individual ASVs, colored according to their phylum. Network parameters shown include the number of nodes (N) and edges (L). Network robustness under targeted removal was evaluated by sequentially removing highly connected nodes. “Remaining species” represents the proportion of nodes/species retained after each removal step. A steeper slope indicates greater network fragility. The panels are organized by biogeomorphic condition (Poor, Moderate, and Good). (**a**) 18S-18S interaction networks and robustness analysis constructed using only coral reefs characteristic 18S rRNA gene-derived ASVs. (**b**) 16S-16S interaction networks and robustness analysis constructed using only coral reef characteristic 16S rRNA gene-derived ASVs. (**c**) 16S-18S (cross-kingdom) interaction networks and robustness analysis constructed using both coral reefs characteristic 16S and 18S ASVs.

The number of nodes in the 18S-18S sub-network, for example, went up steadily from 354 in the Poor state to 447 in the Good state. However, its edges and average degree were highest in the Moderate state before declining, and its average clustering coefficient went from 0.90 to 0.79. Due to these modifications, as health conditions improved, the potential interactions among eukaryotic microorganisms transitioned from dense, localized connections to more selective, sparse ones (Fig. 4a; Table S3). The 16S-16S sub-network attained its peak complexity in the Moderate state. But in the Good state, the number of edges decreased significantly by 39.1%, while the average path length increased significantly from 3.66 to 7.99. These results showed that the structure was optimized by cutting out unnecessary connections, even though these modifications might have made network transfer less efficient (Fig. 4b; Table S3). The 16S-18S cross-kingdom interaction networks were the biggest, and their connectivity trend was the same as that of the whole network. The higher sensitivity of the 16S-18S cross-domain network may reflect functional complementarity between microbial eukaryotes and prokaryotes. 18S rRNA gene-derived microorganisms can influence carbon input, organic matter release, and food-web structure, whereas 16S rRNA gene-derived microorganisms are closely involved in DOM utilization, organic matter degradation, and nutrient regeneration (54, 55). Therefore, 16S-18S associations may better capture coupled changes in carbon flow, nutrient cycling, trophic interactions, and metabolite exchange across reef biogeomorphy states (47, 56). These results meant that interactions between different kingdoms were the main cause of changes in the structure of the whole network, which shows how important these interactions are for the health of an ecosystem (Fig. 4c; Table S3).

Stability analysis made the link between network structure and function even clearer. The Poor state had the weakest sub-networks. It is interesting to note that the Good state network had fewer connections and a less dense structure (lower connectivity), but it was still very stable. The 16S rRNA gene-derived microorganisms network stayed strong even though it had almost 40% fewer edges, which is similar to the Moderate state. The 18S rRNA gene-derived microorganisms network, on the other hand, was strongest in the Good state. This phenomenon corresponded with theories concerning functional redundancy and resilience mechanisms in microbial systems (48, 57), indicating that the preservation of community stability depended not solely on the number of connections but on the structural optimization of interaction networks through the removal of inefficient redundant links and the consolidation of essential functional interactions. These results offer novel insights into the microbial interaction mechanisms within coral reef ecosystems and their responses to environmental changes.

To resolve the topological basis of these stability differences, key nodes were classified via Zi-Pi analysis (Fig. S8). The results showed that ecosystem recovery manifested structurally as an ordered evolution from local centralization to global integration. During the Poor and Moderate stages, network structures were not very complicated, and most nodes were peripheral. In the 18S-16S sub-network, the number of module hubs went from one in the Poor state to three in the Moderate state. This shows that early recovery was marked by the strengthening of local functional units (Fig. S8c). In the Good state, the number of module hubs in the 18S-16S sub-network peaked (five hubs; Fig. S8c), and a single connector appeared for the first time in the 16S-16S and 18S-18S sub-networks, respectively (Fig. S8b and d). The continuous increase in module hubs and the emergence of connectors collectively mark a transition from a structure of relatively independent modules to one of functional integration. This change in topology is an important sign of how well the ecosystem is recovering.

## DISCUSSION

### Reef condition diagnosis beyond richness metrics

Across the South China Sea reef seascape, our results show that reef biogeomorphy structures planktonic microbiomes through spatial reorganization rather than through simple gains or losses of richness. The eigen microstate entropy framework delineated a near-reef influence zone within 588 m in which community composition diverged sharply from off-reef waters, consistent with reefs acting as coupled physical barriers and biogeochemical sources that reshape local dispersal fields and resource landscapes (46, 58, 59). Yet, alpha diversity remained broadly invariant across both distance classes and habitat grades, indicating that richness metrics largely decouple from biogeomorphic state in this system. This apparent stability is most parsimoniously explained by high functional redundancy and rapid taxonomic replacement (57), where sensitive taxa decline under degradation while ecologically similar opportunists occupy vacated niches, preserving total richness while shifting interaction structure and ecological roles (60). The implication is that reef condition is better diagnosed by turnover-sensitive features such as characteristic ASVs and network architecture than by richness-based summaries that underdetect state change.

### State-dependent assembly and a transitional restructuring window

Community assembly analyses further clarify why composition can reorganize without large changes in alpha diversity and why this reorganization is state-dependent and nonlinear. For the reef characteristic taxa, neutral fits were poor, and phylogenetic null models indicated that stochastic processes dominate overall turnover, particularly in the Poor and Good endpoints, where dispersal, drift, and undominated dynamics collectively outweighed selection. This pattern is consistent with a scenario in which habitat filtering first constrains the ecological repertoire that can persist near reefs, after which ecologically similar taxa within those ecological guilds sort largely through stochastic replacement (61). It is interesting to note that determinism grew stronger in the Moderate state for 16S rRNA gene-derived characteristic microorganisms. This suggests a transitional restructuring window in which environmental gradients, resource composition, or biotic feedbacks sharpen selection and temporarily amplify niche differentiation (62). 18S rRNA gene-derived characteristic microorganisms followed a different path. They had stronger deterministic effects when conditions were bad and weaker selection as reefs recovered (63). This is in line with the idea that stress makes environmental conditions more strict for eukaryotic lineages, while recovery makes filtering less strict and allows more randomness. In addition, fraction-specific differences in taxonomic composition, assembly-process contributions, and environmental associations likely reflect size-dependent niche partitioning between larger or particle-associated microorganisms and smaller, predominantly free-living components. For example, *Calanoida* was more prominent in the >3.0 µm fraction, while the environmental correlates of network structure varied among fractions; nevertheless, these associations were generally limited, and the main diversity and assembly patterns remained broadly consistent across size classes (Fig. S9 to S12, Table S5). This cross-fraction consistency indicates that reef biogeomorphic state exerts an ecological influence that extends beyond individual microbial size classes. Together, these results support a two-layer view of reef microbiome dynamics where broad community structure can be strongly filtered at the ecosystem scale, while the reef characteristic subset exhibits high niche overlap that permits drift-dominated turnover except during intermediate restructuring phases.

### From biomarkers to network resilience

The taxa and interaction networks that shift across biogeomorphic states provide a mechanistic bridge between reef form and resilience. The set of 104 state-responsive ASVs captured coherent ecological transitions, including enrichment of detritus-associated heterotrophs and putatively parasitic or saprophytic eukaryotes in degraded reefs, the appearance of calcifying red algal indicators in Moderate reefs consistent with partial retention of reef-framework-associated ecological processes, and the emergence of a fuller trophic structure in healthier reefs. These community patterns coincided with a hump-shaped response in co-occurrence network complexity, with maximal connectivity in Moderate reefs and a sparser but more persistent architecture in Good reefs. This combination argues that recovery is not simply a return to higher interaction density, but a reconfiguration toward streamlined, modular networks with reinforced cross-kingdom coupling and an increased prominence of module hubs and connectors that support system-level integration while pruning redundant links (64, 65). Cross-referencing the 104 state-responsive biomarker ASVs with the state-specific networks revealed that 83 were retained as network nodes. Most of these biomarkers were peripherals (96.3%), indicating that their diagnostic value primarily reflected state-specific ecological responses rather than network-wide structural importance. The emergence of module hubs in Moderate reefs and a connector in Good reefs suggests that a small subset of biomarkers may additionally support within-module cohesion during transitional restructuring and cross-module integration in healthier reefs, respectively. Network-based metrics that capture these role distributions and the balance between local modularity and global connectivity therefore emerge as candidate resilience indicators, complementing taxon-level biomarkers for diagnosing reef condition in rapidly changing tropical seas.

### Conclusion

This study elucidates the adaptive response of microbial communities to different biogeomorphic states of coral reefs in the South China Sea. A microstate entropy-distance approach delineated a near-reef influence zone within 588 m, where community composition and biomarkers differed from off-reef waters despite broadly stable alpha diversity across space and habitat grades, indicating that richness metrics alone underdetect biogeomorphic change. Analyses of 1,162 common reef-characteristic ASVs showed that their turnover is predominantly governed by stochastic processes, while deterministic signals strengthen in the intermediate biogeomorphic state. Co-occurrence networks exhibited maximal complexity under intermediate biogeomorphic conditions but became sparser yet more robust in the healthy state, driven largely by cross-kingdom interactions and increasing module hubs and connectors. Together, these results provide process-relevant microbial indicators and interaction-architecture metrics for diagnosing reef condition and resilience under rapid environmental change.

## Data Availability

The raw sequencing data generated in this study have been deposited in the NCBI BioProject database. The 16S and 18S rRNA gene amplicon data are available under BioProject accession numbers PRJNA1313444, PRJNA1313774, and PRJNA1472955. A detailed list mapping all sample IDs to their corresponding accession codes is provided in Table S4. The script for the data analysis has been uploaded to: https://github.com/Zhengyuelab/Coral-reef.

## SUPPLEMENTAL MATERIAL

Supplemental material aem.01169-26-s0001.pdf Fig. S1 to S12; Tables S1 to S8.

ASM does not own the copyrights to Supplemental Material that may be linked to, or accessed through, an article. The authors have granted ASM a non-exclusive, world-wide license to publish the Supplemental Material files. Please contact the corresponding author directly for reuse.

## References

[1] Galand PE, Ruscheweyh H-J, Salazar G, Hochart C, Henry N, Hume BCC, Oliveira PH, Perdereau A, Labadie K, Belser C, et al.. 2023. Diversity of the Pacific ocean coral reef microbiome. Nat Commun 14:3039. doi:10.1038/s41467-023-38500-x 37264002 PMC10235103

[2] Chen P-Y, Chen C-C, Chu L, McCarl B. 2015. Evaluating the economic damage of climate change on global coral reefs. Global Environmental Change 30:12–20. doi:10.1016/j.gloenvcha.2014.10.011

[3] Reguero BG, Beck MW, Agostini VN, Kramer P, Hancock B. 2018. Coral reefs for coastal protection: a new methodological approach and engineering case study in Grenada. J Environ Manage 210:146–161. doi:10.1016/j.jenvman.2018.01.024 29339333

[4] Morais RA, Smallhorn-West P, Connolly SR, Ngaluafe PF, Malimali S, Halafihi T, Bellwood DR. 2023. Sustained productivity and the persistence of coral reef fisheries. Nat Sustain 6:1199–1209. doi:10.1038/s41893-023-01137-1

[5] Santos HF, Carmo FL, Duarte G, Dini-Andreote F, Castro CB, Rosado AS, van Elsas JD, Peixoto RS. 2014. Climate change affects key nitrogen-fixing bacterial populations on coral reefs. ISME J 8:2272–2279. doi:10.1038/ismej.2014.70 24830827 PMC4992079

[6] Fukunaga A, Burns JHR, Pascoe KH, Kosaki RK. 2022. A remote coral reef shows macroalgal succession following a mass bleaching event. Ecol Indic 142:109175. doi:10.1016/j.ecolind.2022.109175

[7] Perry CT, Murphy GN, Kench PS, Smithers SG, Edinger EN, Steneck RS, Mumby PJ. 2013. Caribbean-wide decline in carbonate production threatens coral reef growth. Nat Commun 4:1402. doi:10.1038/ncomms2409 23360993 PMC3660652

[8] Hughes TP, Kerry JT, Álvarez-Noriega M, Álvarez-Romero JG, Anderson KD, Baird AH, Babcock RC, Beger M, Bellwood DR, Berkelmans R, et al.. 2017. Global warming and recurrent mass bleaching of corals. Nature 543:373–377. doi:10.1038/nature21707 28300113

[9] Dove SG, Brown KT, Van Den Heuvel A, Chai A, Hoegh-Guldberg O. 2020. Ocean warming and acidification uncouple calcification from calcifier biomass which accelerates coral reef decline. Commun Earth Environ 1:55. doi:10.1038/s43247-020-00054-x

[10] Vanwonterghem I, Webster NS. 2020. Coral reef microorganisms in a changing climate. iScience 23:100972. doi:10.1016/j.isci.2020.100972 32208346 PMC7096749

[11] Howard RD, Schul MD, Rodriguez Bravo LM, Altieri AH, Meyer JL. 2023. Shifts in the coral microbiome in response to *in situ* experimental deoxygenation. Appl Environ Microbiol 89:e0057723. doi:10.1128/aem.00577-23 37916820 PMC10686059

[12] Diaz-Pulido G, Barrón C. 2020. CO_2_ enrichment stimulates dissolved organic carbon release in coral reef macroalgae. J Phycol 56:1039–1052. doi:10.1111/jpy.13002 32279320

[13] Wolfe K, Kenyon TM, Mumby PJ. 2021. The biology and ecology of coral rubble and implications for the future of coral reefs. Coral Reefs 40:1769–1806. doi:10.1007/s00338-021-02185-9

[14] Iha C, Dougan KE, Varela JA, Avila V, Jackson CJ, Bogaert KA, Chen Y, Judd LM, Wick R, Holt KE, Pasella MM, Ricci F, Repetti SI, Medina M, Marcelino VR, Chan CX, Verbruggen H. 2021. Genomic adaptations to an endolithic lifestyle in the coral-associated alga *Ostreobium*. Curr Biol 31:1393–1402. doi:10.1016/j.cub.2021.01.018 33548192

[15] Szilagyi Zs, Webster JM, Patterson MA, Hips K, Riding R, Foley M, Humblet M, Yokoyama Y, Liang L, Gischler E, Montaggioni L, Gherardi D, Braga JC. 2020. Controls on the spatio-temporal distribution of microbialite crusts on the Great barrier reef over the past 30,000 years. Mar Geol 429:106312. doi:10.1016/j.margeo.2020.106312

[16] Voolstra CR, Raina J-B, Dörr M, Cárdenas A, Pogoreutz C, Silveira CB, Mohamed AR, Bourne DG, Luo H, Amin SA, Peixoto RS. 2024. The coral microbiome in sickness, in health and in a changing world. Nat Rev Microbiol 22:460–475. doi:10.1038/s41579-024-01015-3 38438489

[17] Glynn VM, de Barros Marangoni LF, Guglielmetti M, Tapia ER, Ali V, Quintero H, Rodriguez Guerra EC, Yuval M, Kline DI, Leray M, Connolly SR, Barrett RDH. 2025. The role of holobiont composition and environmental history in thermotolerance of Tropical Eastern Pacific corals. Curr Biol 35:3048–3063. doi:10.1016/j.cub.2025.05.035 40480235

[18] Xiao J, Wang W, Wang X, Tian P, Niu W. 2022. Recent deterioration of coral reefs in the South China Sea due to multiple disturbances. PeerJ 10:e13634. doi:10.7717/peerj.13634 35910778 PMC9332401

[19] Yu W, Wang W, Yu K, Wang Y, Huang X, Huang R, Liao Z, Xu S, Chen X. 2019. Rapid decline of a relatively high latitude coral assemblage at Weizhou Island, northern South China Sea. Biodivers Conserv 28:3925–3949. doi:10.1007/s10531-019-01858-w

[20] Pascoal F, Tomasino MP, Piredda R, Quero GM, Torgo L, Poulain J, Galand PE, Fuhrman JA, Mitchell A, Tinta T, et al.. 2023. Inter-comparison of marine microbiome sampling protocols. ISME Commun 3:84. doi:10.1038/s43705-023-00278-w 37598259 PMC10439934

[21] Lee C, Song H, Choi Y, Cho A, Marshall J. 2025. Observed multi-decadal increase in the surface ocean’s thermal inertia. Nat Clim Chang 15:308–314. doi:10.1038/s41558-025-02245-w

[22] Muggeo VMR. 2003. Estimating regression models with unknown break-points. Stat Med 22:3055–3071. doi:10.1002/sim.1545 12973787

[23] Can I, Javan GT, Pozhitkov AE, Noble PA. 2014. Distinctive thanatomicrobiome signatures found in the blood and internal organs of humans. J Microbiol Methods 106:1–7. doi:10.1016/j.mimet.2014.07.026 25091187

[24] Dai T, Wen D, Bates CT, Wu L, Guo X, Liu S, Su Y, Lei J, Zhou J, Yang Y. 2022. Nutrient supply controls the linkage between species abundance and ecological interactions in marine bacterial communities. Nat Commun 13:175. doi:10.1038/s41467-021-27857-6 35013303 PMC8748817

[25] Huse SM, Dethlefsen L, Huber JA, Mark Welch D, Relman DA, Sogin ML. 2008. Exploring microbial diversity and taxonomy using SSU rRNA hypervariable tag sequencing. PLoS Genet 4:e1000255. doi:10.1371/journal.pgen.1000255 19023400 PMC2577301

[26] Caporaso JG, Lauber CL, Walters WA, Berg-Lyons D, Lozupone CA, Turnbaugh PJ, Fierer N, Knight R. 2011. Global patterns of 16S rRNA diversity at a depth of millions of sequences per sample. Proc Natl Acad Sci USA 108:4516–4522. doi:10.1073/pnas.1000080107 20534432 PMC3063599

[27] Zhou J, Zhang B-Y, Yu K, Du X-P, Zhu J-M, Zeng Y-H, Cai Z-H. 2020. Functional profiles of phycospheric microorganisms during a marine dinoflagellate bloom. Water Res 173:115554. doi:10.1016/j.watres.2020.115554 32028248

[28] Bolger AM, Lohse M, Usadel B. 2014. Trimmomatic: a flexible trimmer for Illumina sequence data. Bioinformatics 30:2114–2120. doi:10.1093/bioinformatics/btu170 24695404 PMC4103590

[29] Bolyen E, Rideout JR, Dillon MR, Bokulich NA, Abnet CC, Al-Ghalith GA, Alexander H, Alm EJ, Arumugam M, Asnicar F, et al.. 2019. Reproducible, interactive, scalable and extensible microbiome data science using QIIME 2. Nat Biotechnol 37:852–857. doi:10.1038/s41587-019-0209-9 31341288 PMC7015180

[30] Callahan BJ, McMurdie PJ, Rosen MJ, Han AW, Johnson AJA, Holmes SP. 2016. DADA2: high-resolution sample inference from Illumina amplicon data. Nat Methods 13:581–583. doi:10.1038/nmeth.3869 27214047 PMC4927377

[31] Katoh K, Misawa K, Kuma K, Miyata T. 2002. MAFFT: a novel method for rapid multiple sequence alignment based on fast Fourier transform. Nucleic Acids Res 30:3059–3066. doi:10.1093/nar/gkf436 12136088 PMC135756

[32] Price MN, Dehal PS, Arkin AP. 2010. FastTree 2 – approximately maximum-likelihood trees for large alignments. PLoS One 5:e9490. doi:10.1371/journal.pone.0009490 20224823 PMC2835736

[33] Nearing JT, Douglas GM, Hayes MG, MacDonald J, Desai DK, Allward N, Jones CMA, Wright RJ, Dhanani AS, Comeau AM, Langille MGI. 2022. Microbiome differential abundance methods produce different results across 38 datasets. Nat Commun 13:342. doi:10.1038/s41467-022-28034-z 35039521 PMC8763921

[34] R Core Team. 2024. R: a language and environment for statistical computing.

[35] Love MI, Huber W, Anders S. 2014. Moderated estimation of fold change and dispersion for RNA-seq data with DESeq2. Genome Biol 15:550. doi:10.1186/s13059-014-0550-8 25516281 PMC4302049

[36] McMurdie PJ, Holmes S. 2014. Waste not, want not: why rarefying microbiome data is inadmissible. PLoS Comput Biol 10:e1003531. doi:10.1371/journal.pcbi.1003531 24699258 PMC3974642

[37] Breiman L. 2001. Random forests. Mach Learn 45:5–32. doi:10.1023/A:1010933404324

[38] Robin X, Turck N, Hainard A, Tiberti N, Lisacek F, Sanchez J-C, Müller M. 2011. pROC: an open-source package for R and S+ to analyze and compare ROC curves. BMC Bioinformatics 12:77. doi:10.1186/1471-2105-12-77 21414208 PMC3068975

[39] Camacho C, Coulouris G, Avagyan V, Ma N, Papadopoulos J, Bealer K, Madden TL. 2009. BLAST+: architecture and applications. BMC Bioinformatics 10:421. doi:10.1186/1471-2105-10-421 20003500 PMC2803857

[40] Sloan WT, Lunn M, Woodcock S, Head IM, Nee S, Curtis TP. 2006. Quantifying the roles of immigration and chance in shaping prokaryote community structure. Environ Microbiol 8:732–740. doi:10.1111/j.1462-2920.2005.00956.x 16584484

[41] Stegen JC, Lin X, Konopka AE, Fredrickson JK. 2012. Stochastic and deterministic assembly processes in subsurface microbial communities. ISME J 6:1653–1664. doi:10.1038/ismej.2012.22 22456445 PMC3498916

[42] Stegen JC, Lin X, Fredrickson JK, Chen X, Kennedy DW, Murray CJ, Rockhold ML, Konopka A. 2013. Quantifying community assembly processes and identifying features that impose them. ISME J 7:2069–2079. doi:10.1038/ismej.2013.93 23739053 PMC3806266

[43] Wen T, Xie P, Yang S, Niu G, Liu X, Ding Z, Xue C, Liu Y-X, Shen Q, Yuan J. 2022. ggClusterNet: an R package for microbiome network analysis and modularity-based multiple network layouts. Imeta 1:e32. doi:10.1002/imt2.32 38868720 PMC10989811

[44] Csardi G, Nepusz T. 2005. The igraph software package for complex network research. InterJournal Complex Systems.

[45] Dinsdale EA, Pantos O, Smriga S, Edwards RA, Angly F, Wegley L, Hatay M, Hall D, Brown E, Haynes M, Krause L, Sala E, Sandin SA, Thurber RV, Willis BL, Azam F, Knowlton N, Rohwer F. 2008. Microbial ecology of four coral atolls in the Northern Line Islands. PLoS One 3:e1584. doi:10.1371/journal.pone.0001584 18301735 PMC2253183

[46] Comstock J, Nelson CE, James A, Wear E, Baetge N, Remple K, Juknavorian A, Carlson CA. 2022. Bacterioplankton communities reveal horizontal and vertical influence of an Island mass effect. Environ Microbiol 24:4193–4208. doi:10.1111/1462-2920.16092 35691616 PMC9796716

[47] Haas AF, Fairoz MFM, Kelly LW, Nelson CE, Dinsdale EA, Edwards RA, Giles S, Hatay M, Hisakawa N, Knowles B, Lim YW, Maughan H, Pantos O, Roach TNF, Sanchez SE, Silveira CB, Sandin S, Smith JE, Rohwer F. 2016. Global microbialization of coral reefs. Nat Microbiol 1:16042. doi:10.1038/nmicrobiol.2016.42 27572833

[48] Louca S, Polz MF, Mazel F, Albright MBN, Huber JA, O’Connor MI, Ackermann M, Hahn AS, Srivastava DS, Crowe SA, Doebeli M, Parfrey LW. 2018. Function and functional redundancy in microbial systems. Nat Ecol Evol 2:936–943. doi:10.1038/s41559-018-0519-1 29662222

[49] Milke F, Wagner-Doebler I, Wienhausen G, Simon M. 2022. Selection, drift and community interactions shape microbial biogeographic patterns in the Pacific Ocean. ISME J 16:2653–2665. doi:10.1038/s41396-022-01318-4 36115923 PMC9666467

[50] Li J, Chai G, Xiao Y, Li Z. 2023. The impacts of ocean acidification, warming and their interactive effects on coral prokaryotic symbionts. Environ Microbiome 18:49. doi:10.1186/s40793-023-00505-w 37287087 PMC10246149

[51] Remple KL, Silbiger NJ, Quinlan ZA, Fox MD, Kelly LW, Donahue MJ, Nelson CE. 2021. Coral reef biofilm bacterial diversity and successional trajectories are structured by reef benthic organisms and shift under chronic nutrient enrichment. NPJ Biofilms Microbiomes 7:84. doi:10.1038/s41522-021-00252-1 34853316 PMC8636626

[52] Im D-H, Suh H-L. 2019. Evidence for resource partitioning by ontogeny and species in calanoid copepods. Prog Oceanogr 176:102111. doi:10.1016/j.pocean.2019.05.003

[53] Fox JF. 1979. Intermediate-disturbance hypothesis. Science 204:1344–1345. doi:10.1126/science.204.4399.1344 17813173

[54] Seymour JR, Amin SA, Raina J-B, Stocker R. 2017. Zooming in on the phycosphere: the ecological interface for phytoplankton-bacteria relationships. Nat Microbiol 2:17065. doi:10.1038/nmicrobiol.2017.65 28555622

[55] Nelson CE, Wegley Kelly L, Haas AF. 2023. Microbial interactions with dissolved organic matter are central to coral reef ecosystem function and resilience. Ann Rev Mar Sci 15:431–460. doi:10.1146/annurev-marine-042121-080917 36100218

[56] Wild C, Rasheed M, Werner U, Franke U, Johnstone R, Huettel M. 2004. Degradation and mineralization of coral mucus in reef environments. Mar Ecol Prog Ser 267:159–171. doi:10.3354/meps267159

[57] McWilliam M, Hoogenboom MO, Baird AH, Kuo C-Y, Madin JS, Hughes TP. 2018. Biogeographical disparity in the functional diversity and redundancy of corals. Proc Natl Acad Sci USA 115:3084–3089. doi:10.1073/pnas.1716643115 29507193 PMC5866567

[58] Fakhraee M. 2026. Blue carbon ecosystems and coral reefs as coupled nature-based climate solutions. Nat Sustain 9:494–500. doi:10.1038/s41893-026-01768-0

[59] Gove JM, McManus MA, Neuheimer AB, Polovina JJ, Drazen JC, Smith CR, Merrifield MA, Friedlander AM, Ehses JS, Young CW, Dillon AK, Williams GJ. 2016. Near-island biological hotspots in barren ocean basins. Nat Commun 7:10581. doi:10.1038/ncomms10581 26881874 PMC4757766

[60] Glasl B, Bourne DG, Frade PR, Thomas T, Schaffelke B, Webster NS. 2019. Microbial indicators of environmental perturbations in coral reef ecosystems. Microbiome 7:94. doi:10.1186/s40168-019-0705-7 31227022 PMC6588946

[61] Zou J, Guo Y, Zhang A, Shao G, Ma Z, Yu G, Qin C. 2025. Structure and assembly mechanisms of the microbial community on an artificial reef surface, Fangchenggang, China. Appl Microbiol Biotechnol 109:23. doi:10.1007/s00253-025-13415-3 39862282 PMC11762584

[62] Santillan E, Seshan H, Constancias F, Drautz-Moses DI, Wuertz S. 2019. Frequency of disturbance alters diversity, function, and underlying assembly mechanisms of complex bacterial communities. NPJ Biofilms Microbiomes 5:8. doi:10.1038/s41522-019-0079-4 30774969 PMC6370796

[63] Wang S, Gu S, Zhang Y, Deng Y, Qiu W, Sun Q, Zhang T, Wang P, Yan Z. 2024. Microeukaryotic plankton community dynamics under ecological water replenishment: insights from eDNA metabarcoding. Environ Sci Ecotechnol 20:100409. doi:10.1016/j.ese.2024.100409 38572085 PMC10987827

[64] Hernandez-Agreda A, Leggat W, Bongaerts P, Herrera C, Ainsworth TD. 2018. Rethinking the coral microbiome: simplicity exists within a diverse microbial biosphere. mBio 9:e00812-18. doi:10.1128/mBio.00812-18 30301849 PMC6178627

[65] Swain TD, Lax S, Gilbert J, Backman V, Marcelino LA. 2021. A phylogeny-informed analysis of the global coral-symbiodiniaceae interaction network reveals that traits correlated with thermal bleaching are specific to symbiont transmission mode. mSystems 6:e00266-21. doi:10.1128/mSystems.00266-21 33947806 PMC8269218

