# Supplementary material for: Nonlinear response mechanisms of microbial communities to coral reef biogeomorphy in the South China Sea
Source: Appl Environ Microbiol. 2026 Aug 3;92(8):e01169-26. doi: 10.1128/aem.01169-26 (PMC13488313; doi:10.1128/aem.01169-26)
Supplement: Supplementary file 1 [file aem.01169-26-s0001.pdf]

Supplementary Material

Nonlinear Response Mechanisms of Microbial Communities to Coral Reef Biogeomorphy in the South China Sea

Shijie Long[1,a], Zesheng Yang[1,a], Cong Zeng[2], Hongqiang Yang[3,4], Ling Cao[5], Dazhi Wang[1,6], Yali Liu[7,8,9], Qiang Lin[7,8,9], Yue Zheng[1,*]

[1] State Key Laboratory of Marine Environmental Science, College of the Environment and Ecology, Xiamen University, Xiamen 361005, China

[2] School of Oceanography, Shanghai Jiao Tong University, Shanghai 200030, China

[3] State Key Laboratory of Tropical Oceanography, South China Sea Institute of Oceanology, Chinese Academy of Sciences, Guangzhou 510301, China

[4] Nansha Marine Ecological and Environmental Research Station, Chinese Academy of Sciences, Sansha 573100, China

[5] State Key Laboratory of Marine Environmental Science, College of Ocean and Earth Sciences, Xiamen University, Xiamen 361005, China

[6] Southern Marine Science and Engineering Guangdong Laboratory (Guangzhou), Guangzhou 511458, China

[7] CAS Key Laboratory of Tropical Marine Bio-Resources and Ecology, South China Sea Institute of Oceanology, Guangdong Provincial Key Laboratory of Applied Marine Biology, Chinese Academy of Sciences, Guangzhou 510301, China.

[8] Marine Biodiversity and Ecological Evolution Research Center, South China Sea Institute of Oceanology, Chinese Academy of Sciences, Guangzhou 510301, China.

[9] University of Chinese Academy of Sciences, Beijing 100101, China.

** Corresponding author.*

E-mail address: yzheng@xmu.edu.cn (Y. Zheng).

[a] These authors contributed equally to this work.

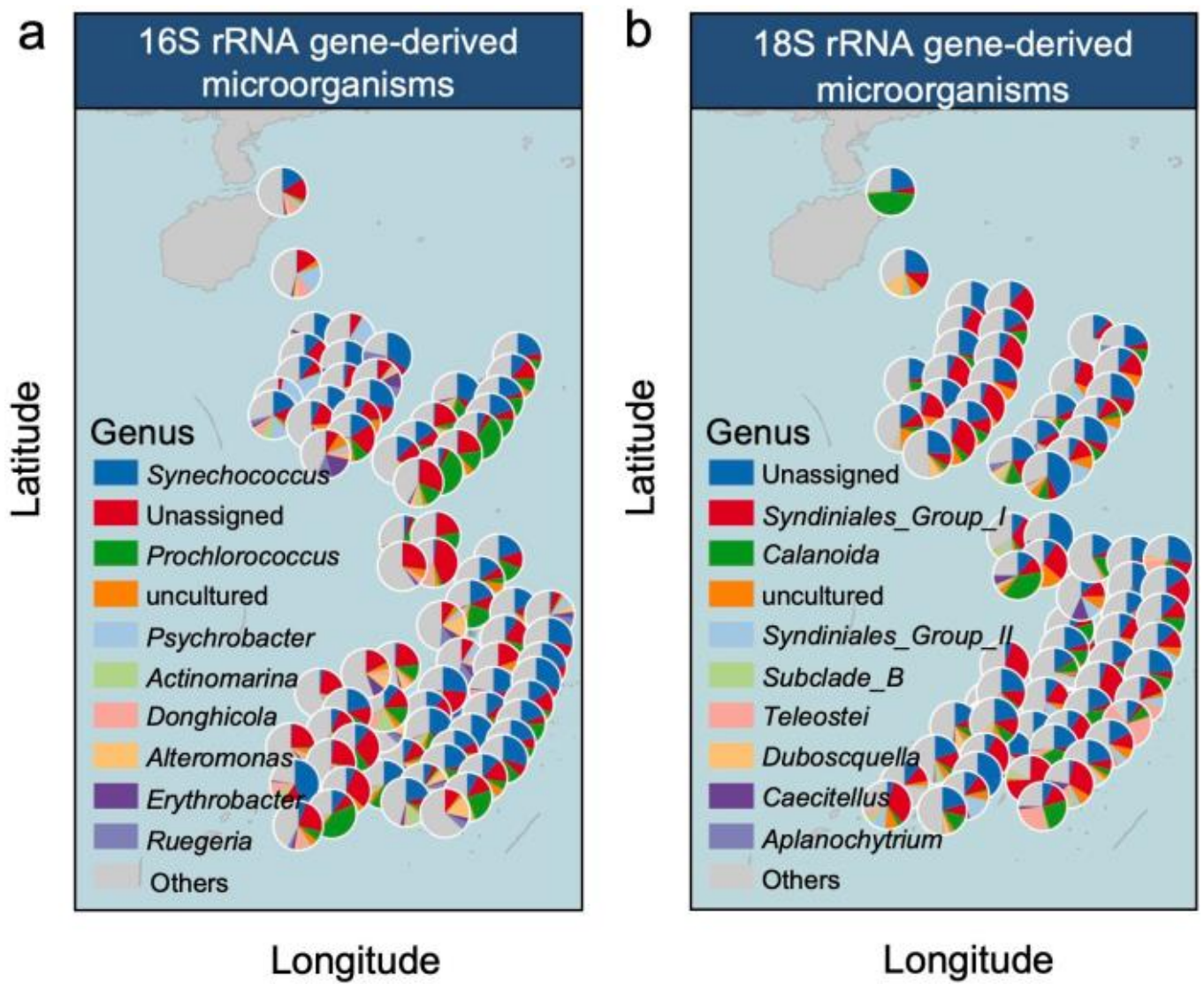

Fig. S1. Spatial distribution patterns of coral reefs microbial communities in the South China Sea. (a, b) Taxonomic composition of the top 10 most abundant 16S rRNA gene-derived microorganisms (a) and 18S rRNA gene-derived microorganisms (b) genera at each sampling site (0.22-3.0 μm size fraction). Each pie chart represents a sampling station, with colors corresponding to different taxa.

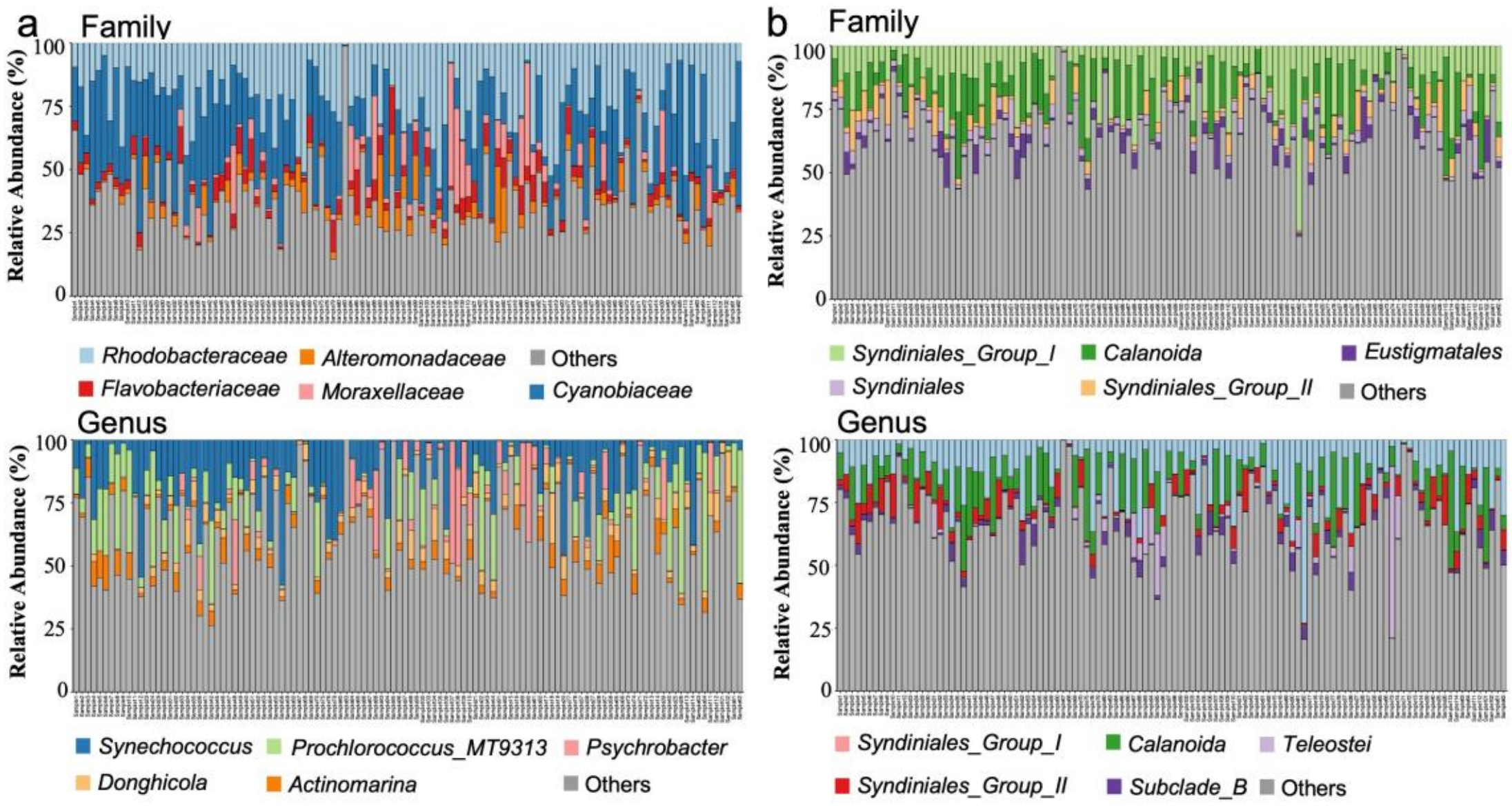

Fig. S2. Taxonomic composition of 16S and 18S rRNA gene-derived microorganisms. (a, b) Stacked bar charts showing the relative abundance of 16S rRNA gene-derived microorganisms (a) and 18S rRNA gene-derived microorganisms (b) at different taxonomic levels (family and genus). Each bar represents an individual sample. Legends highlight the dominant taxa at the corresponding taxonomic level, while rare taxa are merged into the "Others" category (grey). Samples are ordered from left to right according to their distance from the coral reef center.

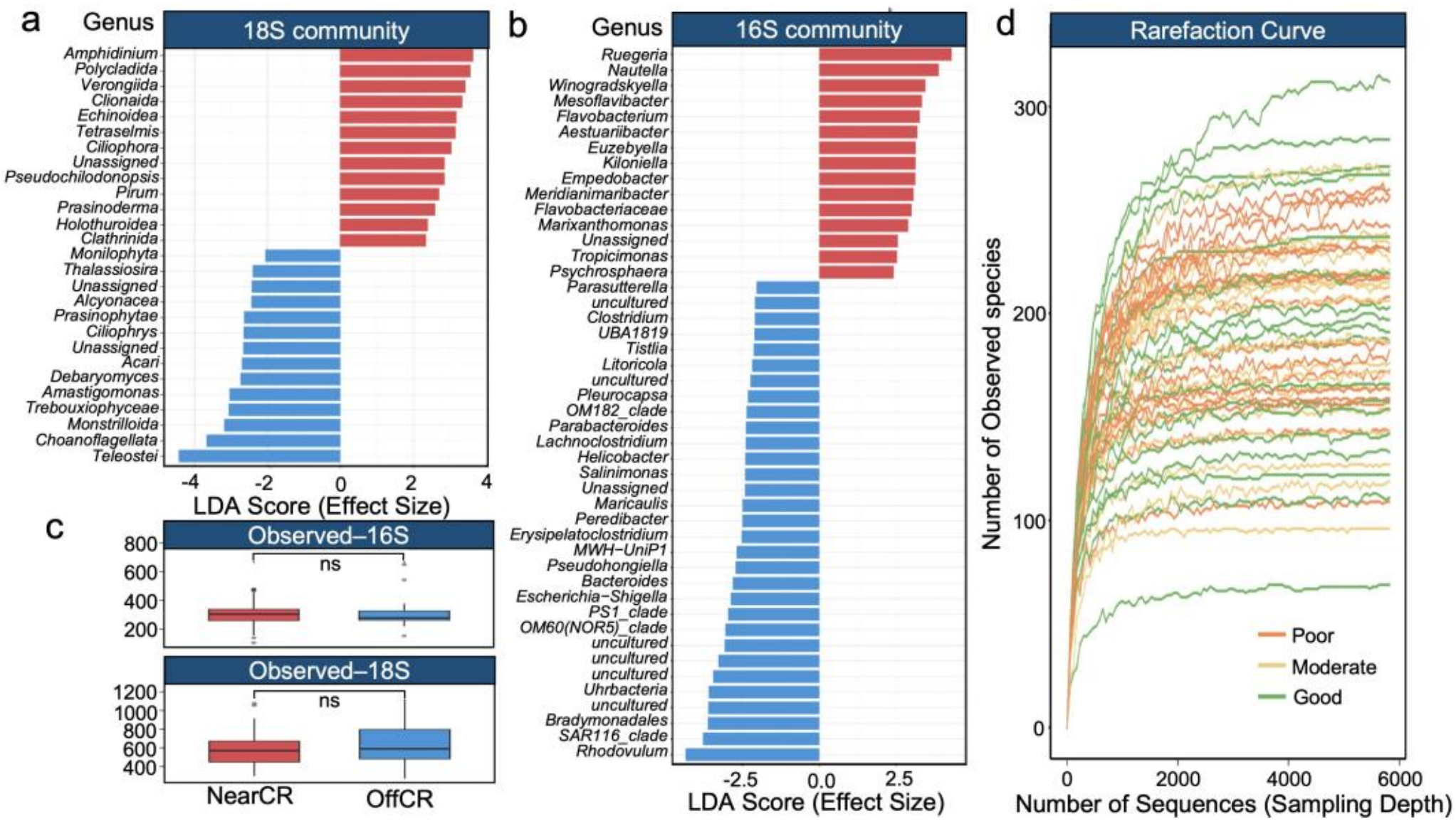

Fig. S3. Characterization of the community structure and diversity of 16S and 18S rRNA gene-derived microorganisms across different zones. LEfSe analysis identifying significantly differentially abundant genera between NearCR and OffCR zones for 18S rRNA gene-derived microorganisms (a) and 16S rRNA gene-derived microorganisms (b). Only biomarkers with an LDA score > 2.0 are shown. Bar length indicates the effect size, with red bars representing microorganisms enriched in the NearCR zone and blue bars representing microorganisms enriched in the OffCR zone. (c) Comparison of Alpha diversity (Observed species) between NearCR and OffCR groups for 16S rRNA gene-derived microorganisms (upper panel) and 18S rRNA gene-derived microorganisms (lower panel). The numbers between boxplots indicate *p*-values from the Wilcoxon rank-sum test. (d) The figure shows rarefaction curves of Observed species diversity among the Poor, Moderate, and Good groups.

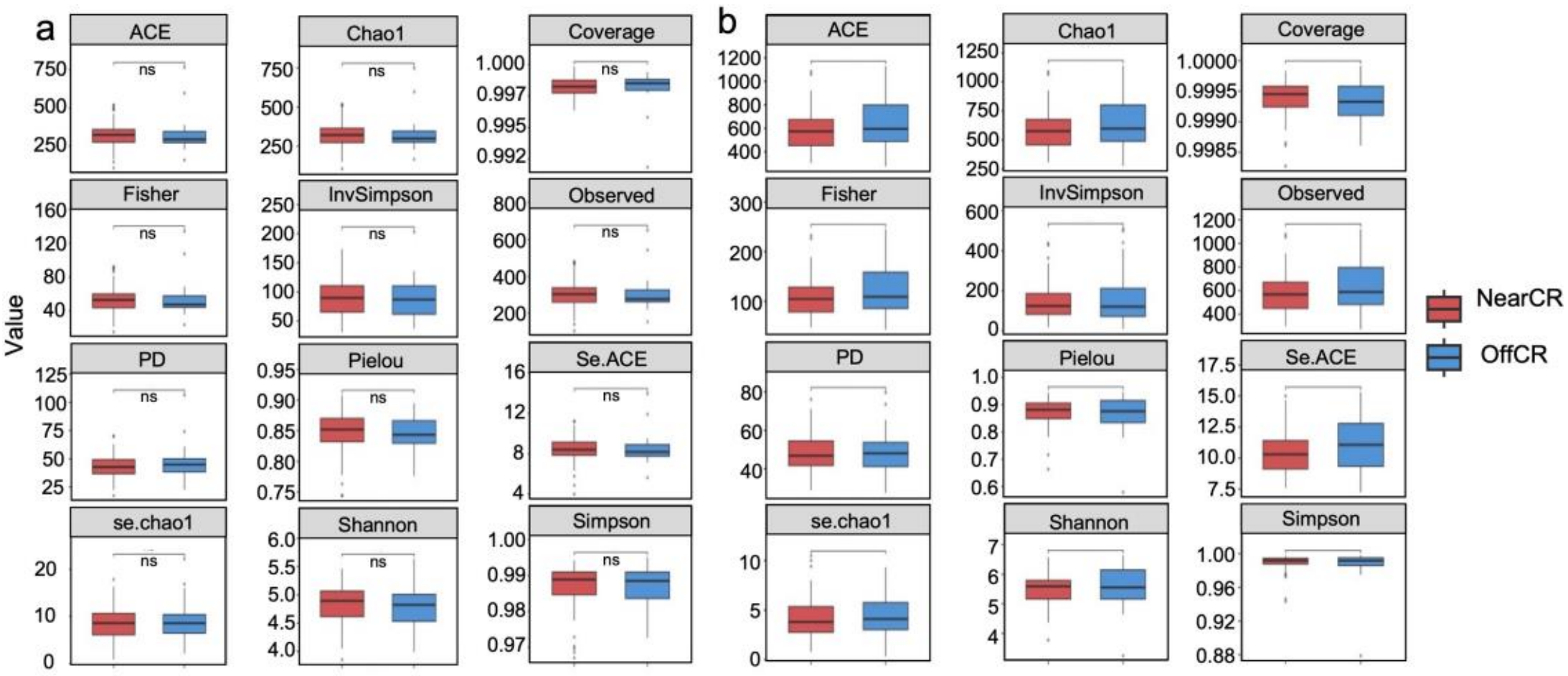

Fig. S4. Comparison of Alpha diversity indices for 16S/18S rRNA gene-derived microorganisms between NearCR and OffCR groups. Boxplots comparing the Alpha diversity indices of (a) 16S rRNA gene-derived microorganisms and (b) 18S rRNA gene-derived microorganisms between the NearCR (red) and OffCR (blue) groups. Each subplot represents a distinct diversity metric, including species richness (e.g., ACE, Chao1, Observed), evenness (e.g., Pielou), and composite diversity indices (e.g., Shannon, Simpson, Fisher). The thick line within each box indicates the median, box boundaries represent the interquartile range (IQR), and whiskers extend to the furthest point within 1.5 × IQR from the box; data points beyond this range are considered outliers. Statistical significance of differences between groups was determined using the Wilcoxon rank-sum test, indicating no significant differences in Alpha diversity for any metric (ns: $p > 0.05$).

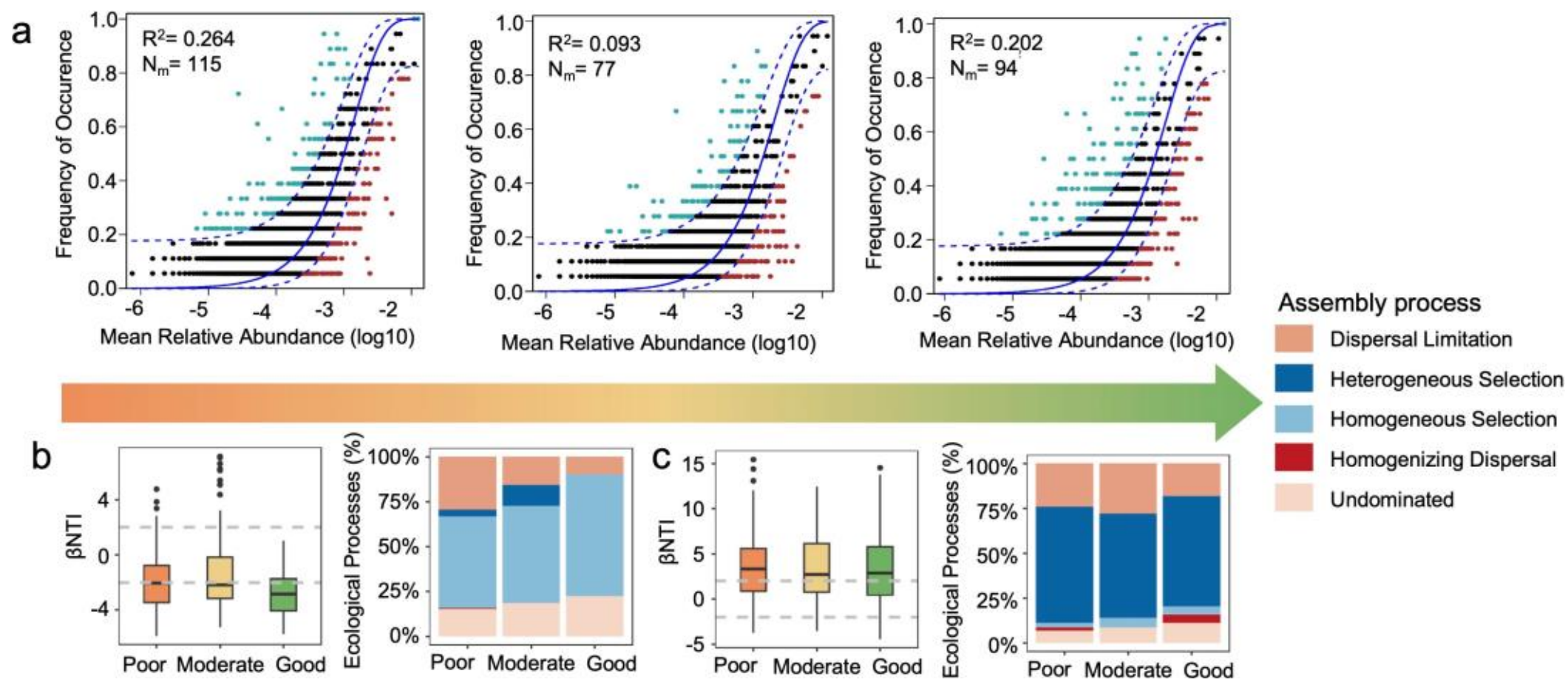

Fig. S5. Assembly mechanisms of the total microbial community in coral reefs ecosystems. (a) Neutral Community Model (NCM) fitting results for the total microbial community. This model evaluates the role of stochastic processes (dispersal and ecological drift) in community assembly across three coral reefs health conditions: Poor, Moderate, and Good. The x-axis represents the mean relative abundance of species (log_{10}-transformed), and the y-axis represents the frequency of occurrence. The solid blue line indicates the model prediction, dashed lines represent the 95% confidence intervals, and black dots represent individual ASVs. R^2 indicates the goodness-of-fit, and Nm denotes the estimated migration rate. (b, c) Phylogenetic null model analysis quantifying the contribution of different ecological processes to community assembly. Left panels: Boxplots showing the distribution of the β-Nearest Taxon Index (βNTI), distinguishing deterministic processes (|βNTI| > 2) from stochastic processes (|βNTI| <2). Right panels: Stacked bar charts illustrating the relative contribution of five ecological processes: Dispersal Limitation, Heterogeneous Selection, Homogeneous Selection, Homogenizing Dispersal, and Undominated stochastic processes. (b) Total 16S rRNA gene-derived microorganisms; (c) Total 18S rRNA gene-derived microorganisms.

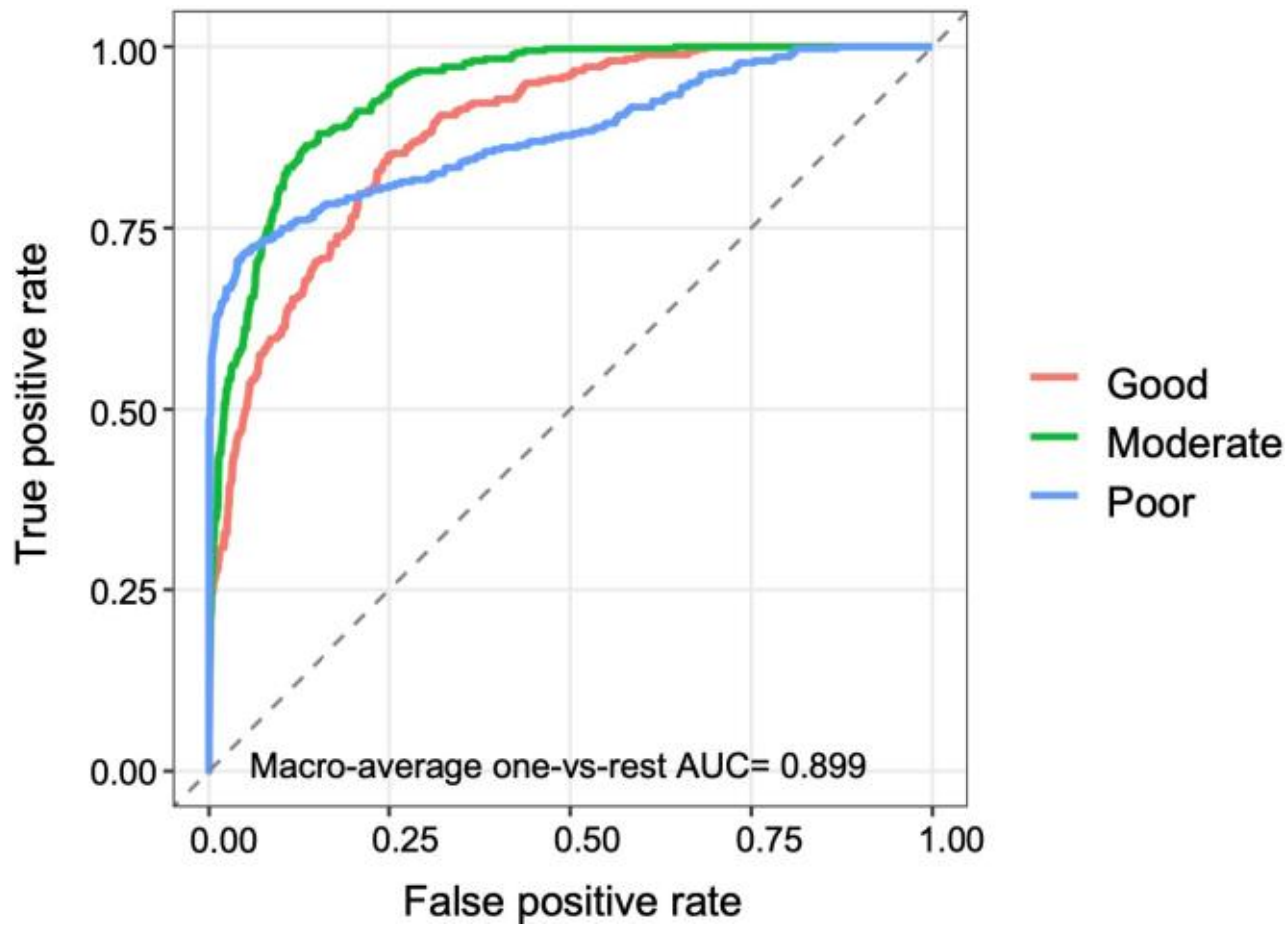

Fig. S6. Random forest-based classification of reef biogeomorphy states. One-vs-rest receiver operating characteristic (ROC) curves showing the performance of the random forest model in classifying Good, Moderate, and Poor reef biogeomorphy states.

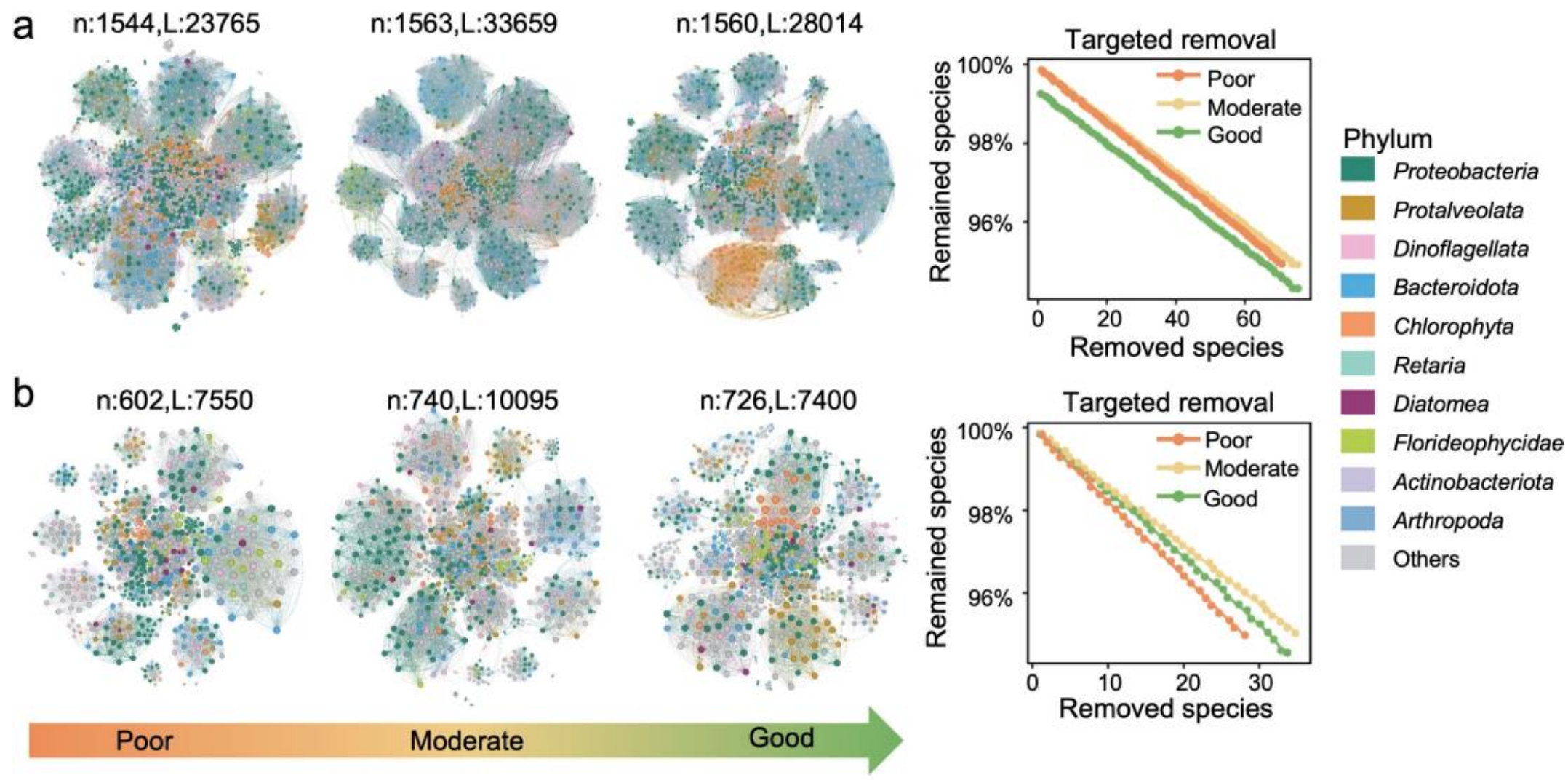

Fig. S7. Co-occurrence networks and stability analysis of total microbial communities versus coral reefs characteristic ASVs. Co-occurrence network diagrams: Nodes represent individual ASVs, colored according to their phylum. Network parameters shown include the number of nodes (n) and edges (L). Robustness was assessed by targeted removal of highly connected nodes. "Remained species" represents the proportion of nodes/species retained after node removal. The panels are organized by coral reefs health status (Poor, Moderate, and Good). (a) Networks and robustness analysis constructed using total ASVs. (b) Networks and robustness analysis constructed using coral reefs characteristic ASVs.

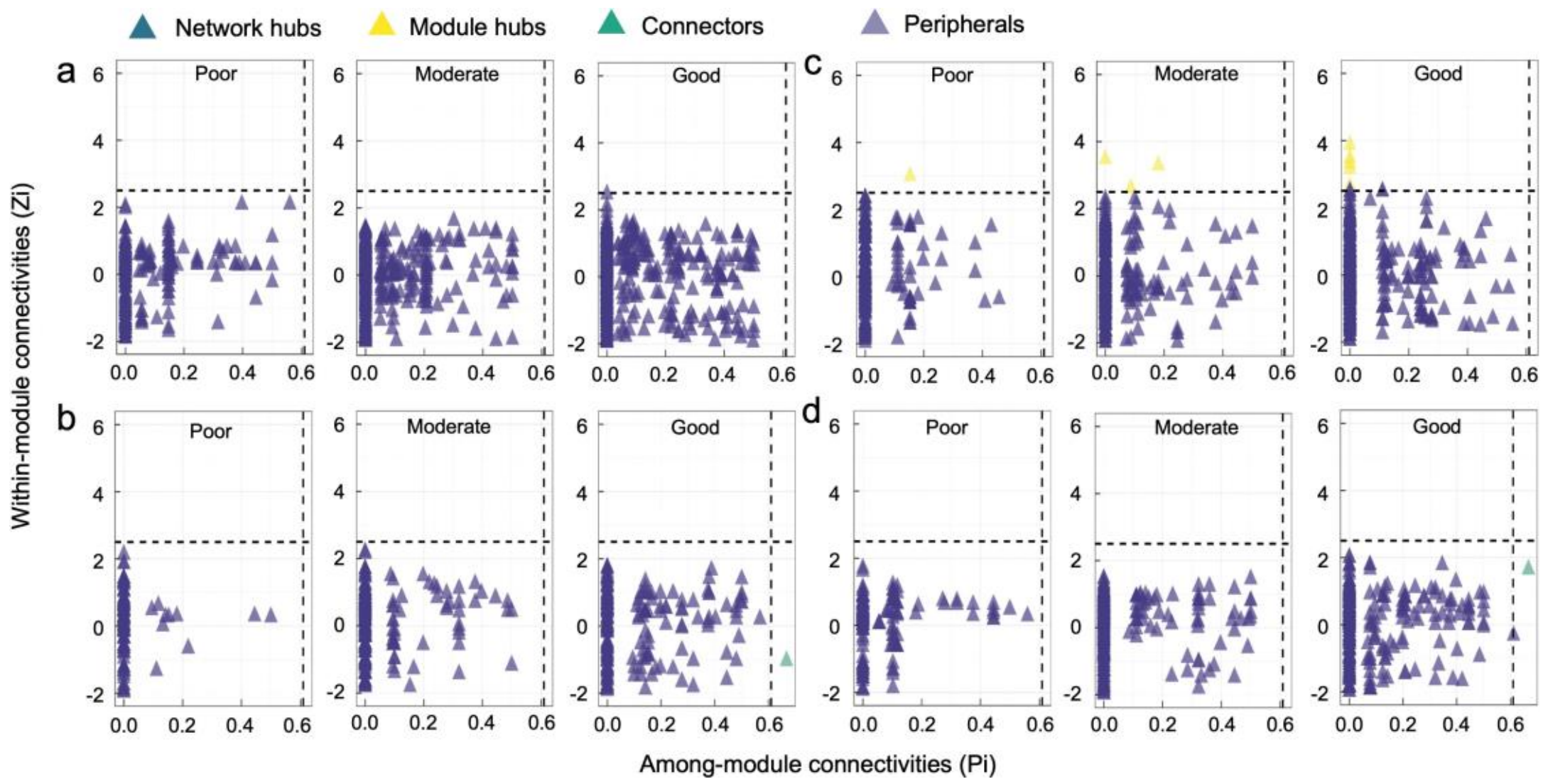

Fig. S8. Topological roles of individual ASVs in microbial co-occurrence networks. Classification of ASV (node) topological roles within the networks based on Zi-Pi values. The topological role of each node is determined jointly by its within-module connectivity (Zi) and among-module connectivity (Pi). Based on standard thresholds (indicated by dashed lines, Zi = 2.5 and Pi = 0.62), nodes are categorized into four classes: (i) Peripherals (Zi ⩽ 2.5, Pi ⩽ 0.62), which have few links to other nodes within their own modules; (ii) Connectors (Zi ⩽ 2.5, Pi > 0.62), which act as bridges between different modules; (iii) Module hubs (Zi > 2.5, Pi ⩽ 0.62), which play key roles within their own modules; (iv) Network hubs (Zi > 2.5, Pi > 0.62), which function as both module hubs and connectors. Rows represent the three coral reefs health conditions: Poor, Moderate, and Good. Columns represent: (a) the overall coral reefs characteristic ASV network, (b) 16S-16S sub-networks, (c) 16S-18S sub-networks, and (d) 18S-18S sub-networks. Different colored triangles denote specific topological roles as indicated by the legend on the right.

To evaluate whether analyzing the two size fractions (>3.0 μm and 0.22-3.0 μm) together affected the main conclusions, we separately analyzed taxonomic composition, alpha diversity, and community assembly processes for 16S and 18S rRNA gene-derived microorganisms in each size fraction. The size-fraction-specific taxonomic patterns were broadly consistent with the combined results. For 16S rRNA gene-derived microorganisms, the dominant families were consistent across size fractions, with *Rhodobacteraceae* and *Cyanobiaceae* dominating both the >3.0 μm and 0.22-3.0 μm fractions (Fig. S9a,b), which was also consistent with their dominance in the combined 16S dataset (Fig. S2a). For 18S rRNA gene-derived microorganisms, *Syndiniales_Group_I* was dominant in both size fractions, while *Calanoida* showed relatively high abundance in the >3.0 μm fraction (Fig. S9c,d), broadly consistent with the combined result (Fig. S2b). Alpha diversity did not differ significantly between NearCR and OffCR groups in either size fraction for both 16S and 18S rRNA gene-derived microorganisms (p > 0.05; Fig. S10), consistent with the combined analysis (Fig. S4). Community assembly analyses also showed comparable patterns between size fractions and combined communities. For 16S rRNA gene-derived microorganisms, homogeneous selection was the main assembly process across reef biogeomorphy states. Its contributions were 55.5%, 55.5%, and 50.6% in the Good, Moderate, and Poor states, respectively, in the >3.0 μm fraction, and 69.4%, 63.8%, and 60.6%, respectively, in the 0.22-3.0 μm fraction. In contrast, the 18S communities were mainly dominated by heterogeneous selection, with contributions of 41.6%, 55.0%, and 47.6% in the Good, Moderate, and Poor states, respectively, in the >3.0 μm fraction, and 66.6%, 52.7%, and 59.3%, respectively, in the 0.22-3.0 μm fraction (Fig. S10). Overall, although the two size fractions represent ecologically distinct microbial components, the size-fraction-specific analyses supported the main conclusions obtained from the combined dataset.

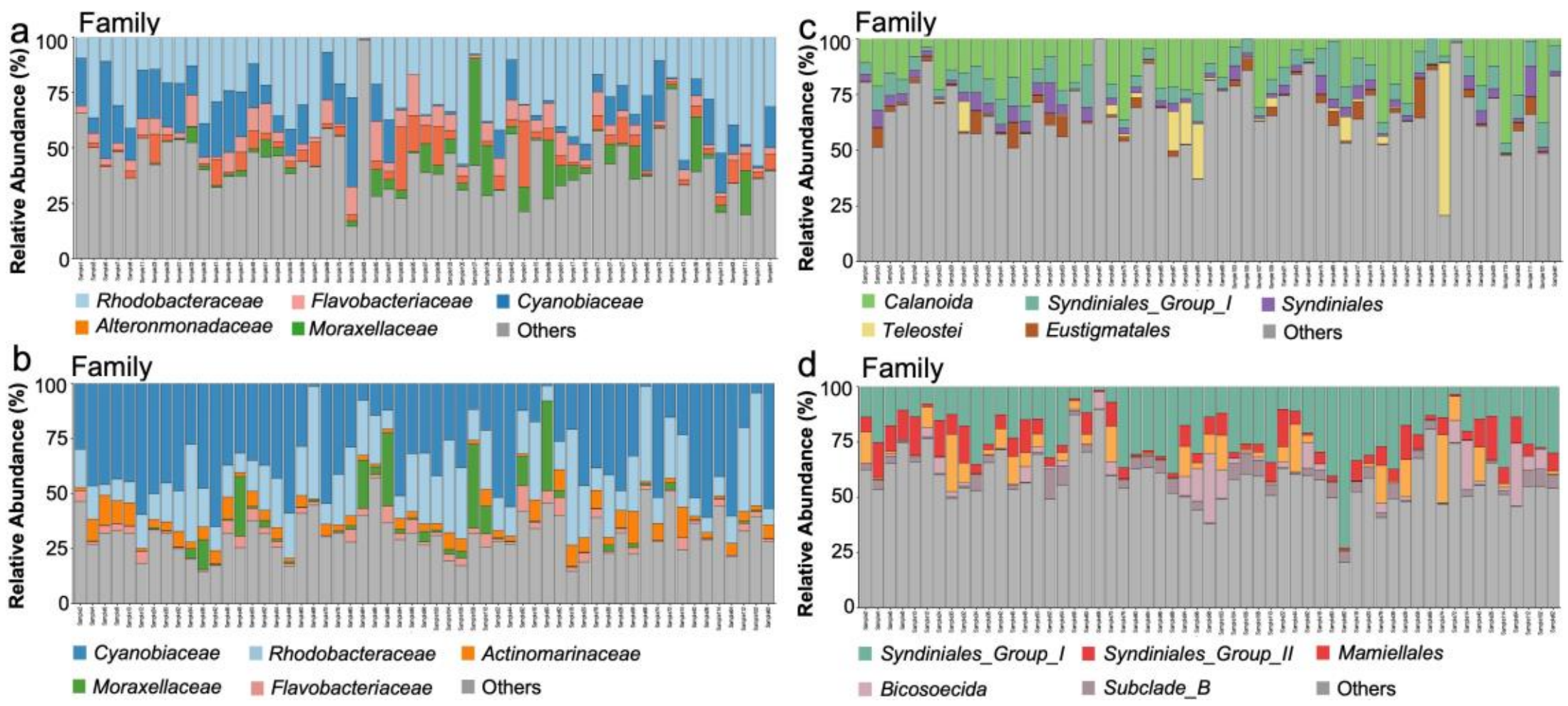

Fig. S9. Taxonomic composition at the family level based on the five most abundant families. (a) 16S rRNA gene-derived microorganisms in the >3.0 μm size fraction. (b) 16S rRNA gene-derived microorganisms in the 0.22-3.0 μm size fraction.

(c) 18S rRNA gene-derived microorganisms in the >3.0 μm size fraction. (d) 18S rRNA gene-derived microorganisms in the 0.22-3.0 μm size fraction.

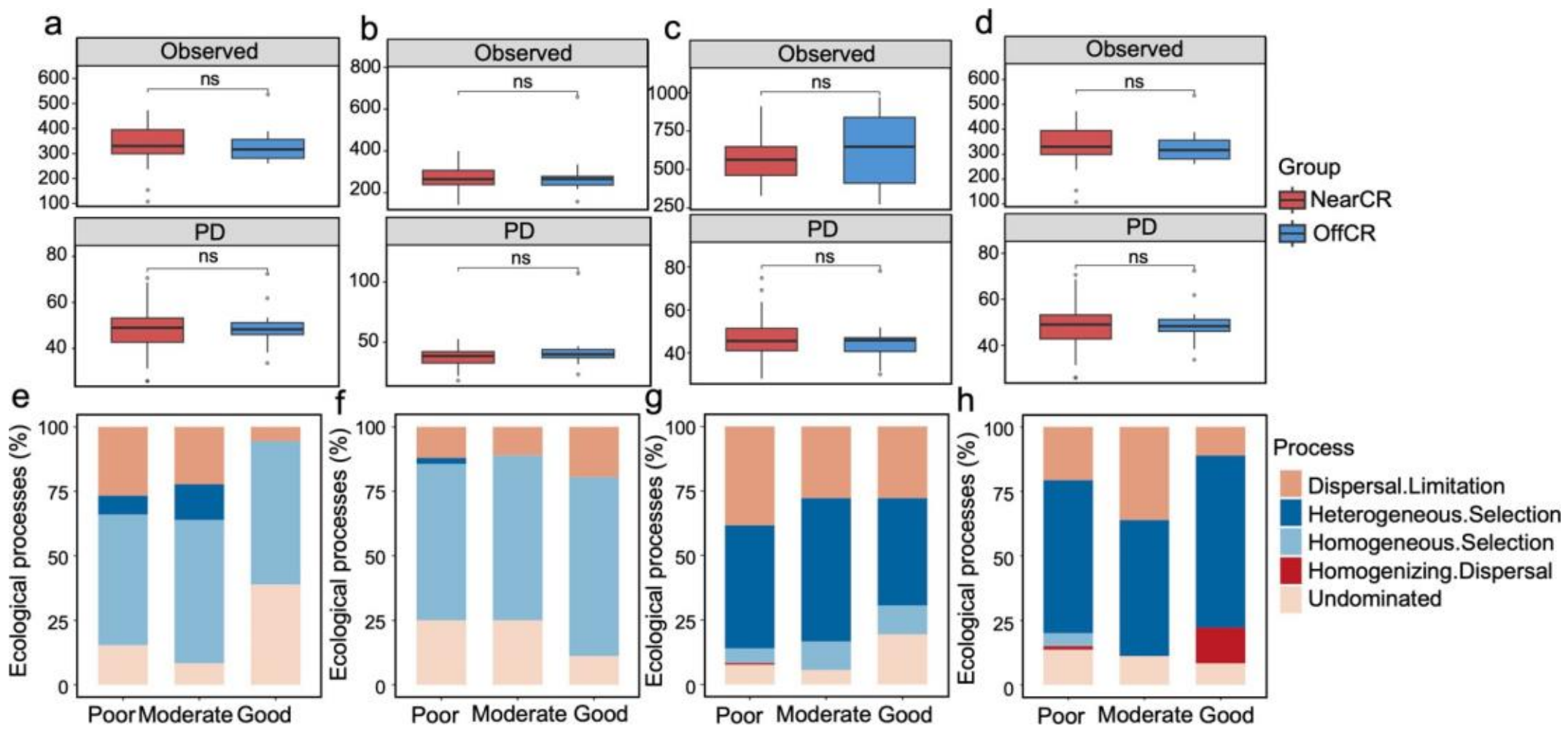

Fig. S10. Alpha diversity and assembly processes across size fractions. Boxplots show Observed richness and Faith's phylogenetic diversity (PD) between NearCR and OffCR groups. Stacked bars show βNTI/RC_{bray}-based assembly processes across reef biogeomorphy states. Panels correspond to 16S-derived microorganisms in the >3.0 μm (a, e) and 0.22-3.0 μm (b, f) fractions, and 18S-derived microorganisms in the >3.0 μm (c, g) and 0.22-3.0 μm (d, h) fractions.

To evaluate the potential influence of environmental gradients, we analyzed environmental differences, CCA, environmental distance-|βNTI| associations, and network-environment correlations. Environmental factors did not differ significantly between NearCR and OffCR groups ($p > 0.05$; Fig. S11a), whereas only conductivity, salinity, and temperature showed significant differences among reef biogeomorphy states (Fig. S11b). CCA analyses indicated that environmental variables explained a limited fraction of variation in microbial community composition. The first two CCA axes accounted for 34.05%, 28.62%, 29.09%, and 32.11% of the variation in the 16S rRNA gene-derived microorganisms of the >3.0 μm and 0.22-3.0 μm fractions and the 18S rRNA gene-derived microorganisms of the >3.0 μm and 0.22-3.0 μm fractions, respectively. The main environmental variables included conductivity, temperature, pH, salinity, DO, and nutrients (Fig. S12a-d). Network-environment correlation analyses showed that most environmental factors were not significantly associated with co-occurrence network edges or nodes (Fig. S12e-g). Significant correlations were detected only between network nodes and DO/conductivity in the 16S communities of the 0.22-3.0 μm fraction, nitrate in the 18S communities of the >3.0 μm fraction, and salinity in the 18S communities of the 0.22-3.0 μm fraction. The relationships between environmental distance and |βNTI| were generally weak across biogeomorphy states ($R^2 < 0.15$). Significant correlations were detected only for 18S rRNA gene-derived microorganisms in the Poor state of the >3.0 μm fraction (p = 0.004) and in the Moderate state of the 0.22-3.0 μm fraction (p = 0.049) (Table S5). Overall, these analyses suggest that the measured environmental factors were associated with microbial community composition, assembly processes, and network topology to some extent, but their explanatory power was limited and insufficient to fully explain the observed microbial variation.

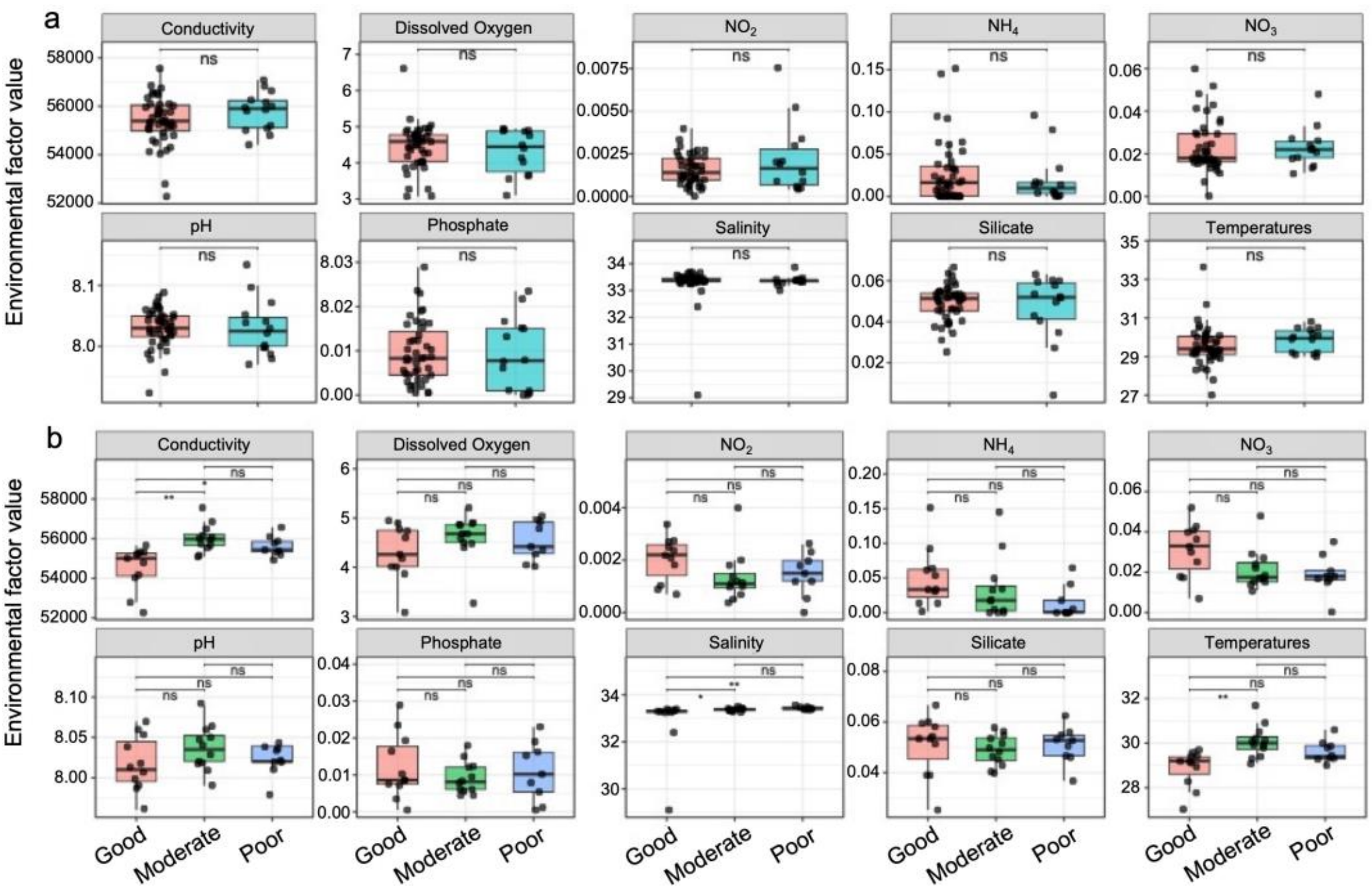

Fig. S11. The environmental factor differences among sampling groups. Differences in environmental factors were assessed using the Wilcoxon test, and *p*-values were adjusted for multiple comparisons using the False Discovery Rate (FDR) method (* $p < 0.05$, * * $p < 0.01$, and * * * $p < 0.001$, "ns" indicates no significant difference). (a) Differences in environmental factors between NearCR and OffCR zones. (b) Differences in environmental factors among Good, Moderate, and Poor states.

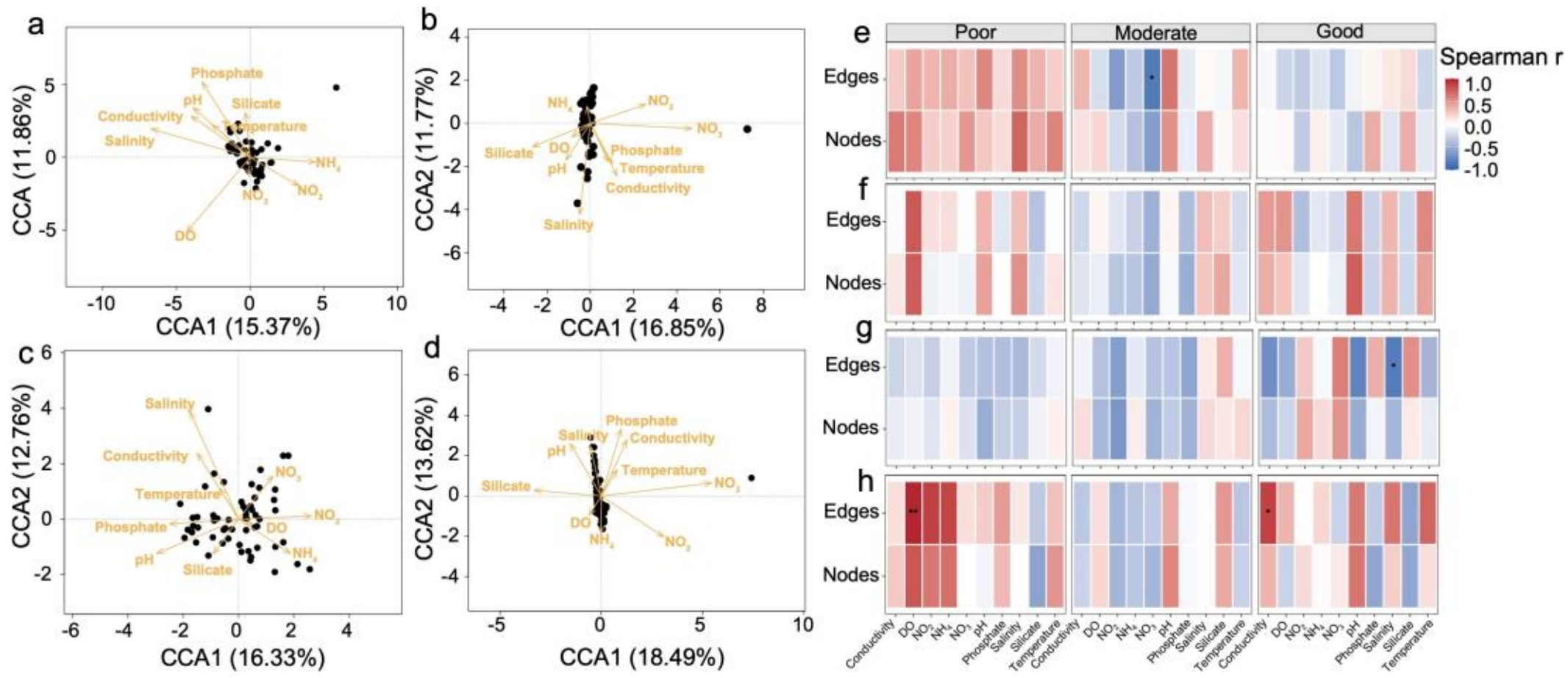

Fig. S12. Environmental effects on microbial community composition and co-occurrence network topology. Canonical correspondence analysis (CCA) showing relationships between environmental variables and microbial community in the >3.0 μm size fraction for 18S (a) and 16S (b) rRNA gene-derived microorganisms, and in the 0.22-3.0 μm size fraction for 18S (c) and 16S (d) rRNA gene-derived microorganisms. Spearman correlation heatmaps showing associations between environmental factors and co-occurrence network topological parameters in the >3.0 μm size fraction for 18S (e) and 16S (f), and in the 0.22-3.0 μm size fraction for 18S (g) and 16S (h) rRNA gene-derived microorganisms.

Table S1. ASVs exhibiting significant differential abundance across different coral reefs biogeomorphic conditions.

ASV	Biogeomorphic status	Kingdom
532d136f9a8d872152b9cea1313d71d4	Poor	Bacteria
9f07019f0dc762200061f1a788a51622	Poor	Eukaryota
0a4582de2144a3bea1fd2daf016e9f02	Poor	Bacteria
89c9d49742a0557041c80f6e7dca11c6	Poor	Eukaryota
f601542559aad29f09809ef063b81db2	Poor	Eukaryota
0966bffa57df96d215055844d5cb3674	Poor	Eukaryota
c4e0466f252a9aeb282f467237ba4d41	Poor	Eukaryota
b2455a6d0438fd0ec43cc55967ade484	Poor	Eukaryota
2a1178f19c9cf4f9317f7c497fd616c2	Poor	Bacteria
7d7ff9a4344aeae9c63e66a48f569539	Poor	Bacteria
ccf23da8fab968d12f7564715eb75ba6	Poor	Bacteria
b85e4e4d785acaa2e912acebb6093130	Poor	Bacteria
dcc539eb6f4ff4251cc93f7c3edee6d8	Poor	Eukaryota
71d4e8c8e7e1602343439b414e4c820d	Poor	Eukaryota
c848874b4820a1b7b124a716bac4a76d	Poor	Eukaryota
8829af98022073fd8c41bc92012d37ac	Poor	Bacteria
8dd7674bdf7be1f8f95b8803c93e600d	Poor	Eukaryota
1c6296d507bfe50ac1b4529b8cac9788	Poor	Eukaryota
6efb91eb350f6e6660cba779a3fafbfc	Poor	Eukaryota
e1fec6de78f010be68720bd5a89347c6	Poor	Bacteria
740ef17916f3a7bc6dcda00fc1184eca	Poor	Bacteria
6e8b41905a2849c42f6c3e4559da5bbc	Poor	Eukaryota
a3cafb18a552b3afc659f15c850cf26f	Moderate	Bacteria
6d5cc74e2bbd281728a9ad639ebd2bf6	Moderate	Bacteria
cdc53eda556d1a7de7c16ab19981b44e	Moderate	Eukaryota
cdf7360bdf4848920c96d8efc542bde6	Moderate	Bacteria
4a2f8d0d2d24ed1ee6ddeba7f0dca854	Moderate	Eukaryota
d7b84830bbe6e75437fc6abad7460bdb	Moderate	Bacteria
17be7b60f937048913ba7e94415f34cd	Moderate	Eukaryota
2ec7430832dcc8af187385953682ab0d	Moderate	Bacteria
e184003b1be168c3f2d89e9aafb9e5c2	Moderate	Bacteria
2cf3104ae09cd480bbbe0edee150e451	Moderate	Eukaryota
1ca66d2a01bd84a218aac334cf6297ff	Moderate	Bacteria
f749a23e153fc2fa2c13e45378be7f45	Moderate	Bacteria
226de50f50e96234c7524089e81baeff	Moderate	Bacteria
22b90c405a00630044da3a77e601200d	Moderate	Eukaryota
f7fb7a5b7d385c435bf9afb713d027a8	Moderate	Bacteria
db2d4735fe9d51cd5499f968d31a53ab	Moderate	Bacteria
b4c8df31a57909b85c88ae1502b84300	Moderate	Bacteria

d5975f4c3d764de97fb91a4b3d1d0ab8	Moderate	Eukaryota
8d41ee71850253ecb2d1c26bf480043a	Moderate	Bacteria
9b1583d588b76e8902abb4a78c94a472	Moderate	Eukaryota
df6e60d22de2453598bc2f4102b7c79f	Moderate	Bacteria
eab50b21dc644ef1ec61c8f3a6539954	Moderate	Bacteria
eaf9e446449999839491aa9443374f89	Moderate	Bacteria
894e3e89a139c16009aa0b36f8d3af8d	Moderate	Bacteria
8912c39456bcfa1551e9c733def9ce65	Moderate	Bacteria
2f526c55d3e08a30a003312432aeda01	Moderate	Bacteria
e4f403934b23b2ff04ad646ce738f2fa	Moderate	Eukaryota
38d38b57121115c02e5463562a11d4ca	Moderate	Bacteria
1bee1ce28e9e700cc68decf5ce46fe77	Moderate	Bacteria
949d83edca450cb796aff58a44d1813f	Moderate	Bacteria
64287e0f8cf71a98f70ca55d4eb38aa0	Moderate	Bacteria
a860fb750c0b03a14bd60851ef5e15c5	Moderate	Bacteria
0b7d8a0ded1424be8e0e0d301d2dfaec	Moderate	Eukaryota
aa07268d23456882211f0173c026d6f9	Moderate	Bacteria
f8e2ddfd4a1a0ab97f0417545efcf5d3	Moderate	Bacteria
188ad90857cce7192ccb5d46e19dca63	Moderate	Eukaryota
e518b90fad967696e1b8694b01775db6	Moderate	Bacteria
9c91ec0e95c79b0f390809061cee26bb	Moderate	Bacteria
3ed24f00a937de5be6862a3fe75f54dc	Moderate	Bacteria
5bb288e6d87b40c87c16bb5702c929a7	Moderate	Bacteria
7439ec0d2659ec876463f51e5bd4b721	Good	Eukaryota
3ced3a637968acaaa513c0c2aa682928	Good	Eukaryota
0580c3bc2a9e89358876db5faceded50	Good	Bacteria
5e38278a9bb73a54b2bec9e12c7f1f4c	Good	Eukaryota
9f6ab43faf82c5dff185e9f3c60c35e4	Good	Bacteria
2f9f93474cb235e09f125f07dcd254af	Good	Eukaryota
fbfd4846bb96dea8a66d790d8e863e18	Good	Eukaryota
b367659ae29474a392661e373860071e	Good	Eukaryota
43799820da690ca957f69adb82d298fb	Good	Bacteria
c7891752eb63ed43fd187af1192532d4	Good	Eukaryota
c61bb4de5f14ca5ffc241ce2315ec2a4	Good	Eukaryota
93a752c0e990ed68f7e1d6ffd7568ab8	Good	Bacteria
cec0c4e58a46b6f5fa3a24e7af66c775	Good	Bacteria
8f16a29c0fc76b5986682e86ab3592dc	Good	Eukaryota
636b653f0d96d7b180576b2463080b11	Good	Eukaryota
5b1e7858c08d37b2fdf2dcda4487245d	Good	Bacteria
5f4ce9014abe6861f29857ceae8bb20d	Good	Bacteria
964d0a09771fdc13042c8690c4f5006a	Good	Bacteria
e445dc4935a9326cf1fe812d504f5e37	Good	Bacteria
0d7b975447da19e5e7028f1740f577dd	Good	Bacteria

fd3d17b4ff1a69e944337a455b9c6ec7	Good	Bacteria
be23553ef22c93dfd828735ebe5c21e0	Good	Bacteria
55b15e28c36117236d45569fe885379a	Good	Bacteria
d42702ad5f31f559d6b1f328671cebde	Good	Bacteria
8837bcb00265a965ad81f57446942a2a	Good	Eukaryota
a8c3696df1b4c943265a59fc3650dda2	Good	Bacteria
bbb4c461424b0ced4c055f8c6a9fc892	Good	Bacteria
ced5c7c82fbe8f014f772a6bbb7be03c	Good	Bacteria
046f10e11d1be5010ce486bca51d3bec	Good	Bacteria
4e28fbe4ff557b9a92ca09448366204d	Good	Eukaryota
b3f74a2e1bd30955529ddca7b9e0fa37	Good	Bacteria
63fd03bf355d4084a14bc55d038408b7	Good	Bacteria
69eb895e9cb8a0af18f8f2cfc233705b	Good	Bacteria
e64e2e91e0bf451aa20bf737a863e6ba	Good	Bacteria
29f7d2f86d8469201b0732bb31c94796	Good	Bacteria
e162b25a736fd139c1ccc548c5a146e7	Good	Eukaryota
8a90520fc3b5c0cbba0e66a6287b6c5f	Good	Eukaryota
a0aeaf8aeeb1020ff672897749c27eb7	Good	Eukaryota
6375b705f5765d52600b5306d706cbd5	Good	Bacteria
219f1d4ac9cfc3b82190b51953e74c18	Good	Bacteria
5f37d851449ab52f8555043136c1d1a0	Good	Eukaryota
69b8ad4c38aa471f137f87225b9653e6	Good	Eukaryota

Table S2. Biogeomorphic state prediction of new reefs based on feature ASVs.

Sample	Original biogeomorphic status	Predicted biogeomorphic status
Sample1	Poor	Moderate
Sample2	Good	Good
Sample3	Moderate	Moderate
Sample4	Poor	Poor
Sample5	Poor	Poor
Sample6	Moderate	Poor
Sample7	Poor	Poor
Sample8	Poor	Poor
Sample9	Poor	Poor
Sample10	Moderate	Poor
Sample11	Poor	Poor
Sample12	Poor	Poor
Sample13	Poor	Poor
Sample14	Poor	Poor

Table S3. Topological properties of different microbial co-occurrence networks.

Group	18S-18S			16S-16S			16S-18S			All		
	Poor	Moderate	Good	Poor	Moderate	Good	Poor	Moderate	Good	Poor	Moderate	Good
Num edges(L)	2992.00	3696.00	3209.00	1404.00	2012.00	1225.00	3406.00	4367.00	3173.00	7550.00	10095.00	7400.00
Num pos edges	2992.00	3696.00	3209.00	1404.00	2012.00	1225.00	0.00	0.00	0.00	7550.00	10095.00	7400.00
Num neg edges	0.00	0.00	0.00	0.00	0.00	0.00	0.00	0.00	0.00	0.00	0.00	0.00
Num vertices(n)	354.00	422.00	447.00	244.00	304.00	271.00	577.00	686.00	591.00	602.00	740.00	726.00
Connectance (edge_density)	0.05	0.04	0.03	0.05	0.04	0.03	0.02	0.02	0.02	0.04	0.04	0.03
Average degree (Average K)	16.90	17.52	14.36	11.51	13.24	9.04	11.81	12.73	10.74	25.08	27.28	20.39
Average path length	4.82	6.96	6.16	3.66	7.83	7.99	3.28	9.68	8.14	6.90	5.57	5.51
Diameter	14.55	17.84	16.11	11.11	19.38	24.35	10.00	27.00	26.00	17.08	13.76	13.92
Edge connectivity	0.00	0.00	0.00	0.00	0.00	0.00	0.00	0.00	0.00	0.00	0.00	0.00
Mean clustering coefficient (Average.CC)	0.90	0.85	0.79	0.89	0.82	0.77	0.00	0.00	0.00	0.89	0.83	0.78
Clusters	11.00	5.00	7.00	13.00	10.00	6.00	12.00	6.00	8.00	6.00	5.00	5.00
Centralization degree	0.06	0.05	0.04	0.04	0.06	0.04	0.04	0.03	0.04	0.05	0.03	0.04
Centralization betweenness	0.09	0.26	0.12	0.06	0.27	0.26	0.02	0.18	0.11	0.28	0.09	0.10
Centralization closeness	1.23	1.49	1.63	1.00	1.50	1.63	1.23	1.62	1.50	1.53	1.67	1.67
RM	4.11	3.88	3.21	2.87	3.09	2.49	/	/	/	5.60	6.08	4.99

Table S4. Details of sequencing samples and their NCBI accession numbers

Accession	Study	Bioproject_accession	Biosample_accession	Sample_name
SRR35209157	SRP615730	PRJNA1313444	SAMN50897254	Bacteria_BE708-09M0001
SRR35209156	SRP615730	PRJNA1313444	SAMN50897255	Bacteria_BE708-09M0002
SRR35209152	SRP615730	PRJNA1313444	SAMN50897258	Bacteria_BE708-09M0005
SRR35209151	SRP615730	PRJNA1313444	SAMN50897259	Bacteria_BE708-09M0006
SRR35208957	SRP615730	PRJNA1313444	SAMN50897262	Bacteria_BE708-09M0009
SRR35208956	SRP615730	PRJNA1313444	SAMN50897263	Bacteria_BE708-09M0010
SRR35208953	SRP615730	PRJNA1313444	SAMN50897266	Bacteria_BE708-09M0013
SRR35208951	SRP615730	PRJNA1313444	SAMN50897267	Bacteria_BE708-09M0014
SRR35208948	SRP615730	PRJNA1313444	SAMN50897270	Bacteria_BE708-09M0017
SRR35208947	SRP615730	PRJNA1313444	SAMN50897271	Bacteria_BE708-09M0018
SRR35208944	SRP615730	PRJNA1313444	SAMN50897274	Bacteria_BE708-09M0021
SRR35208943	SRP615730	PRJNA1313444	SAMN50897275	Bacteria_BE708-09M0022
SRR35208939	SRP615730	PRJNA1313444	SAMN50897278	Bacteria_BE708-09M0025
SRR35208938	SRP615730	PRJNA1313444	SAMN50897279	Bacteria_BE708-09M0026
SRR35208935	SRP615730	PRJNA1313444	SAMN50897282	Bacteria_BE708-09M0029
SRR35208934	SRP615730	PRJNA1313444	SAMN50897283	Bacteria_BE708-09M0030
SRR35208931	SRP615730	PRJNA1313444	SAMN50897286	Bacteria_BE708-09M0033
SRR35208929	SRP615730	PRJNA1313444	SAMN50897287	Bacteria_BE708-09M0034
SRR35208926	SRP615730	PRJNA1313444	SAMN50897290	Bacteria_BE708-09M0037
SRR35208765	SRP615730	PRJNA1313444	SAMN50897291	Bacteria_BE708-09M0038
SRR35208762	SRP615730	PRJNA1313444	SAMN50897294	Bacteria_BE708-09M0041
SRR35208761	SRP615730	PRJNA1313444	SAMN50897295	Bacteria_BE708-09M0042
SRR35208757	SRP615730	PRJNA1313444	SAMN50897298	Bacteria_BE708-09M0045
SRR35208756	SRP615730	PRJNA1313444	SAMN50897299	Bacteria_BE708-09M0046
SRR35208753	SRP615730	PRJNA1313444	SAMN50897302	Bacteria_BE708-09M0049
SRR35208752	SRP615730	PRJNA1313444	SAMN50897303	Bacteria_BE708-09M0050
SRR35209324	SRP615730	PRJNA1313444	SAMN50897306	Bacteria_BE708-09M0053
SRR35209322	SRP615730	PRJNA1313444	SAMN50897307	Bacteria_BE708-09M0054
SRR35209319	SRP615730	PRJNA1313444	SAMN50897310	Bacteria_BE708-09M0057
SRR35209318	SRP615730	PRJNA1313444	SAMN50897311	Bacteria_BE708-09M0058
SRR35209315	SRP615730	PRJNA1313444	SAMN50897314	Bacteria_BE708-09M0061
SRR35209314	SRP615730	PRJNA1313444	SAMN50897315	Bacteria_BE708-09M0062
SRR35209310	SRP615730	PRJNA1313444	SAMN50897318	Bacteria_BE708-09M0065
SRR35209309	SRP615730	PRJNA1313444	SAMN50897319	Bacteria_BE708-09M0066
SRR35208923	SRP615730	PRJNA1313444	SAMN50897322	Bacteria_BE708-09M0069
SRR35208922	SRP615730	PRJNA1313444	SAMN50897323	Bacteria_BE708-09M0070
SRR35208919	SRP615730	PRJNA1313444	SAMN50897326	Bacteria_BE708-09M0073
SRR35208917	SRP615730	PRJNA1313444	SAMN50897327	Bacteria_BE708-09M0074
SRR35208914	SRP615730	PRJNA1313444	SAMN50897330	Bacteria_BE708-09M0077
SRR35208913	SRP615730	PRJNA1313444	SAMN50897331	Bacteria_BE708-09M0078
SRR35208910	SRP615730	PRJNA1313444	SAMN50897334	Bacteria_BE708-09M0081

SRR35208909	SRP615730	PRJNA1313444	SAMN50897335	Bacteria_BE708-09M0082
SRR35208905	SRP615730	PRJNA1313444	SAMN50897338	Bacteria_BE708-09M0085
SRR35208904	SRP615730	PRJNA1313444	SAMN50897339	Bacteria_BE708-09M0086
SRR35208901	SRP615730	PRJNA1313444	SAMN50897342	Bacteria_BE708-09M0089
SRR35208900	SRP615730	PRJNA1313444	SAMN50897343	Bacteria_BE708-09M0090
SRR35208899	SRP615730	PRJNA1313444	SAMN50897344	Bacteria_BE708-09M0091
SRR35208898	SRP615730	PRJNA1313444	SAMN50897345	Bacteria_BE708-09M0092
SRR35208893	SRP615730	PRJNA1313444	SAMN50897348	Bacteria_BE708-09M0095
SRR35208892	SRP615730	PRJNA1313444	SAMN50897349	Bacteria_BE708-09M0096
SRR35208891	SRP615730	PRJNA1313444	SAMN50897350	Bacteria_BE708-09M0097
SRR35208890	SRP615730	PRJNA1313444	SAMN50897351	Bacteria_BE708-09M0098
SRR35208887	SRP615730	PRJNA1313444	SAMN50897354	Bacteria_BE708-09M0101
SRR35208886	SRP615730	PRJNA1313444	SAMN50897355	Bacteria_BE708-09M0102
SRR35208882	SRP615730	PRJNA1313444	SAMN50897358	Bacteria_BE708-09M0105
SRR35208881	SRP615730	PRJNA1313444	SAMN50897359	Bacteria_BE708-09M0106
SRR35208878	SRP615730	PRJNA1313444	SAMN50897362	Bacteria_BE708-09M0109
SRR35208877	SRP615730	PRJNA1313444	SAMN50897363	Bacteria_BE708-09M0110
SRR35208874	SRP615730	PRJNA1313444	SAMN50897366	Bacteria_BE708-09M0113
SRR35208872	SRP615730	PRJNA1313444	SAMN50897367	Bacteria_BE708-09M0114
SRR35208871	SRP615730	PRJNA1313444	SAMN50897368	Bacteria_BE708-09M0115
SRR35208870	SRP615730	PRJNA1313444	SAMN50897369	Bacteria_BE708-09M0116
SRR35208869	SRP615730	PRJNA1313444	SAMN50897370	Bacteria_BE708-09M0117
SRR35208868	SRP615730	PRJNA1313444	SAMN50897371	Bacteria_BE708-09M0118
SRR35208867	SRP615730	PRJNA1313444	SAMN50897372	Bacteria_BE708-09M0119
SRR35208866	SRP615730	PRJNA1313444	SAMN50897373	Bacteria_BE708-09M0120
SRR35208865	SRP615730	PRJNA1313444	SAMN50897374	Bacteria_BE708-09M0121
SRR35208864	SRP615730	PRJNA1313444	SAMN50897375	Bacteria_BE708-09M0122
SRR35208863	SRP615730	PRJNA1313444	SAMN50897376	Bacteria_BE708-09M0123
SRR35209148	SRP615730	PRJNA1313444	SAMN50897377	Bacteria_BE708-09M0124
SRR35209147	SRP615730	PRJNA1313444	SAMN50897378	Bacteria_BE708-09M0125
SRR35209146	SRP615730	PRJNA1313444	SAMN50897379	Bacteria_BE708-09M0126
SRR35209145	SRP615730	PRJNA1313444	SAMN50897380	Bacteria_BE708-09M0127
SRR35209144	SRP615730	PRJNA1313444	SAMN50897381	Bacteria_BE708-09M0128
SRR35209143	SRP615730	PRJNA1313444	SAMN50897382	Bacteria_BE708-09M0129
SRR35209142	SRP615730	PRJNA1313444	SAMN50897383	Bacteria_BE708-09M0130
SRR35209141	SRP615730	PRJNA1313444	SAMN50897384	Bacteria_BE708-09M0131
SRR35209140	SRP615730	PRJNA1313444	SAMN50897385	Bacteria_BE708-09M0132
SRR35209139	SRP615730	PRJNA1313444	SAMN50897386	Bacteria_BE708-09M0133
SRR35209137	SRP615730	PRJNA1313444	SAMN50897387	Bacteria_BE708-09M0134
SRR35209136	SRP615730	PRJNA1313444	SAMN50897388	Bacteria_BE708-09M0135
SRR35209135	SRP615730	PRJNA1313444	SAMN50897389	Bacteria_BE708-09M0136
SRR35209134	SRP615730	PRJNA1313444	SAMN50897390	Bacteria_BE708-09M0137
SRR35209133	SRP615730	PRJNA1313444	SAMN50897391	Bacteria_BE708-09M0138

SRR35209132	SRP615730	PRJNA1313444	SAMN50897392	Bacteria_BE708-09M0139
SRR35209131	SRP615730	PRJNA1313444	SAMN50897393	Bacteria_BE708-09M0140
SRR35209130	SRP615730	PRJNA1313444	SAMN50897394	Bacteria_BE708-09M0141
SRR35209129	SRP615730	PRJNA1313444	SAMN50897395	Bacteria_BE708-09M0142
SRR35209128	SRP615730	PRJNA1313444	SAMN50897396	Bacteria_BE708-09M0143
SRR35209126	SRP615730	PRJNA1313444	SAMN50897397	Bacteria_BE708-09M0144
SRR35209125	SRP615730	PRJNA1313444	SAMN50897398	Bacteria_BE708-09M0145
SRR35209124	SRP615730	PRJNA1313444	SAMN50897399	Bacteria_BE708-09M0146
SRR35209123	SRP615730	PRJNA1313444	SAMN50897400	Bacteria_BE708-09M0147
SRR35209122	SRP615730	PRJNA1313444	SAMN50897401	Bacteria_BE708-09M0148
SRR35209121	SRP615730	PRJNA1313444	SAMN50897402	Bacteria_BE708-09M0149
SRR35209120	SRP615730	PRJNA1313444	SAMN50897403	Bacteria_BE708-09M0150
SRR35209119	SRP615730	PRJNA1313444	SAMN50897404	Bacteria_BE708-09M0151
SRR35209118	SRP615730	PRJNA1313444	SAMN50897405	Bacteria_BE708-09M0152
SRR35209117	SRP615730	PRJNA1313444	SAMN50897406	Bacteria_BE708-09M0153
SRR35209211	SRP615730	PRJNA1313444	SAMN50897407	Bacteria_BE708-09M0154
SRR35209210	SRP615730	PRJNA1313444	SAMN50897408	Bacteria_BE708-09M0155
SRR35209209	SRP615730	PRJNA1313444	SAMN50897409	Bacteria_BE708-09M0156
SRR35209208	SRP615730	PRJNA1313444	SAMN50897410	Bacteria_BE708-09M0157
SRR35209207	SRP615730	PRJNA1313444	SAMN50897411	Bacteria_BE708-09M0158
SRR35209206	SRP615730	PRJNA1313444	SAMN50897412	Bacteria_BE708-09M0159
SRR35209205	SRP615730	PRJNA1313444	SAMN50897413	Bacteria_BE708-09M0160
SRR35209204	SRP615730	PRJNA1313444	SAMN50897414	Bacteria_BE708-09M0161
SRR35209203	SRP615730	PRJNA1313444	SAMN50897415	Bacteria_BE708-09M0162
SRR35209202	SRP615730	PRJNA1313444	SAMN50897416	Bacteria_BE708-09M0163
SRR35209200	SRP615730	PRJNA1313444	SAMN50897417	Bacteria_BE708-09M0164
SRR35209199	SRP615730	PRJNA1313444	SAMN50897418	Bacteria_BE708-09M0165
SRR35209198	SRP615730	PRJNA1313444	SAMN50897419	Bacteria_BE708-09M0166
SRR35209197	SRP615730	PRJNA1313444	SAMN50897420	Bacteria_BE708-09M0167
SRR35209196	SRP615730	PRJNA1313444	SAMN50897421	Bacteria_BE708-09M0168
SRR35222201	SRP616507	PRJNA1313774	SAMN50908319	Unknown_BE708-08M0001
SRR35222200	SRP616507	PRJNA1313774	SAMN50908320	Unknown_BE708-08M0002
SRR35222196	SRP616507	PRJNA1313774	SAMN50908323	Unknown_BE708-08M0005
SRR35222195	SRP616507	PRJNA1313774	SAMN50908324	Unknown_BE708-08M0006
SRR35222192	SRP616507	PRJNA1313774	SAMN50908327	Unknown_BE708-08M0009
SRR35222191	SRP616507	PRJNA1313774	SAMN50908328	Unknown_BE708-08M0010
SRR35221836	SRP616507	PRJNA1313774	SAMN50908331	Unknown_BE708-08M0013
SRR35221834	SRP616507	PRJNA1313774	SAMN50908332	Unknown_BE708-08M0014
SRR35221830	SRP616507	PRJNA1313774	SAMN50908335	Unknown_BE708-08M0017
SRR35221829	SRP616507	PRJNA1313774	SAMN50908336	Unknown_BE708-08M0018
SRR35221826	SRP616507	PRJNA1313774	SAMN50908339	Unknown_BE708-08M0021
SRR35221825	SRP616507	PRJNA1313774	SAMN50908340	Unknown_BE708-08M0022
SRR35221821	SRP616507	PRJNA1313774	SAMN50908343	Unknown_BE708-08M0025

SRR35221820	SRP616507	PRJNA1313774	SAMN50908344	Unknown_BE708-08M0026
SRR35221817	SRP616507	PRJNA1313774	SAMN50908347	Unknown_BE708-08M0029
SRR35221816	SRP616507	PRJNA1313774	SAMN50908348	Unknown_BE708-08M0030
SRR35221813	SRP616507	PRJNA1313774	SAMN50908351	Unknown_BE708-08M0033
SRR35221811	SRP616507	PRJNA1313774	SAMN50908352	Unknown_BE708-08M0034
SRR35222171	SRP616507	PRJNA1313774	SAMN50908355	Unknown_BE708-08M0037
SRR35221808	SRP616507	PRJNA1313774	SAMN50908356	Unknown_BE708-08M0038
SRR35222189	SRP616507	PRJNA1313774	SAMN50908359	Unknown_BE708-08M0041
SRR35222188	SRP616507	PRJNA1313774	SAMN50908360	Unknown_BE708-08M0042
SRR35222184	SRP616507	PRJNA1313774	SAMN50908363	Unknown_BE708-08M0045
SRR35222183	SRP616507	PRJNA1313774	SAMN50908364	Unknown_BE708-08M0046
SRR35222180	SRP616507	PRJNA1313774	SAMN50908367	Unknown_BE708-08M0049
SRR35222179	SRP616507	PRJNA1313774	SAMN50908368	Unknown_BE708-08M0050
SRR35222176	SRP616507	PRJNA1313774	SAMN50908371	Unknown_BE708-08M0053
SRR35222174	SRP616507	PRJNA1313774	SAMN50908372	Unknown_BE708-08M0054
SRR35221925	SRP616507	PRJNA1313774	SAMN50908375	Unknown_BE708-08M0057
SRR35222170	SRP616507	PRJNA1313774	SAMN50908376	Unknown_BE708-08M0058
SRR35222167	SRP616507	PRJNA1313774	SAMN50908379	Unknown_BE708-08M0061
SRR35222166	SRP616507	PRJNA1313774	SAMN50908380	Unknown_BE708-08M0062
SRR35222162	SRP616507	PRJNA1313774	SAMN50908383	Unknown_BE708-08M0065
SRR35222161	SRP616507	PRJNA1313774	SAMN50908384	Unknown_BE708-08M0066
SRR35222158	SRP616507	PRJNA1313774	SAMN50908387	Unknown_BE708-08M0069
SRR35221933	SRP616507	PRJNA1313774	SAMN50908388	Unknown_BE708-08M0070
SRR35221930	SRP616507	PRJNA1313774	SAMN50908391	Unknown_BE708-08M0073
SRR35221928	SRP616507	PRJNA1313774	SAMN50908392	Unknown_BE708-08M0074
SRR35221905	SRP616507	PRJNA1313774	SAMN50908395	Unknown_BE708-08M0077
SRR35221924	SRP616507	PRJNA1313774	SAMN50908396	Unknown_BE708-08M0078
SRR35221921	SRP616507	PRJNA1313774	SAMN50908399	Unknown_BE708-08M0081
SRR35221920	SRP616507	PRJNA1313774	SAMN50908400	Unknown_BE708-08M0082
SRR35221916	SRP616507	PRJNA1313774	SAMN50908403	Unknown_BE708-08M0085
SRR35221915	SRP616507	PRJNA1313774	SAMN50908404	Unknown_BE708-08M0086
SRR35221912	SRP616507	PRJNA1313774	SAMN50908407	Unknown_BE708-08M0089
SRR35221911	SRP616507	PRJNA1313774	SAMN50908408	Unknown_BE708-08M0090
SRR35221910	SRP616507	PRJNA1313774	SAMN50908409	Unknown_BE708-08M0091
SRR35221909	SRP616507	PRJNA1313774	SAMN50908410	Unknown_BE708-08M0092
SRR35221884	SRP616507	PRJNA1313774	SAMN50908413	Unknown_BE708-08M0095
SRR35221883	SRP616507	PRJNA1313774	SAMN50908414	Unknown_BE708-08M0096
SRR35221904	SRP616507	PRJNA1313774	SAMN50908415	Unknown_BE708-08M0097
SRR35221903	SRP616507	PRJNA1313774	SAMN50908416	Unknown_BE708-08M0098
SRR35221900	SRP616507	PRJNA1313774	SAMN50908419	Unknown_BE708-08M0101
SRR35221899	SRP616507	PRJNA1313774	SAMN50908420	Unknown_BE708-08M0102
SRR35221895	SRP616507	PRJNA1313774	SAMN50908423	Unknown_BE708-08M0105
SRR35221894	SRP616507	PRJNA1313774	SAMN50908424	Unknown_BE708-08M0106

SRR35221891	SRP616507	PRJNA1313774	SAMN50908427	Unknown_BE708-08M0109
SRR35221890	SRP616507	PRJNA1313774	SAMN50908428	Unknown_BE708-08M0110
SRR35221871	SRP616507	PRJNA1313774	SAMN50908431	Unknown_BE708-08M0113
SRR35221805	SRP616507	PRJNA1313774	SAMN50908432	Unknown_BE708-08M0114
SRR35221882	SRP616507	PRJNA1313774	SAMN50908433	Unknown_BE708-08M0115
SRR35221881	SRP616507	PRJNA1313774	SAMN50908434	Unknown_BE708-08M0116
SRR35221880	SRP616507	PRJNA1313774	SAMN50908435	Unknown_BE708-08M0117
SRR35221879	SRP616507	PRJNA1313774	SAMN50908436	Unknown_BE708-08M0118
SRR35221878	SRP616507	PRJNA1313774	SAMN50908437	Unknown_BE708-08M0119
SRR35221877	SRP616507	PRJNA1313774	SAMN50908438	Unknown_BE708-08M0120
SRR35221876	SRP616507	PRJNA1313774	SAMN50908439	Unknown_BE708-08M0121
SRR35221875	SRP616507	PRJNA1313774	SAMN50908440	Unknown_BE708-08M0122
SRR35221874	SRP616507	PRJNA1313774	SAMN50908441	Unknown_BE708-08M0123
SRR35221872	SRP616507	PRJNA1313774	SAMN50908442	Unknown_BE708-08M0124
SRR35221792	SRP616507	PRJNA1313774	SAMN50908443	Unknown_BE708-08M0125
SRR35221791	SRP616507	PRJNA1313774	SAMN50908444	Unknown_BE708-08M0126
SRR35221790	SRP616507	PRJNA1313774	SAMN50908445	Unknown_BE708-08M0127
SRR35221789	SRP616507	PRJNA1313774	SAMN50908446	Unknown_BE708-08M0128
SRR35221788	SRP616507	PRJNA1313774	SAMN50908447	Unknown_BE708-08M0129
SRR35221787	SRP616507	PRJNA1313774	SAMN50908448	Unknown_BE708-08M0130
SRR35221786	SRP616507	PRJNA1313774	SAMN50908449	Unknown_BE708-08M0131
SRR35221785	SRP616507	PRJNA1313774	SAMN50908450	Unknown_BE708-08M0132
SRR35221784	SRP616507	PRJNA1313774	SAMN50908451	Unknown_BE708-08M0133
SRR35221782	SRP616507	PRJNA1313774	SAMN50908452	Unknown_BE708-08M0134
SRR35221804	SRP616507	PRJNA1313774	SAMN50908453	Unknown_BE708-08M0135
SRR35221803	SRP616507	PRJNA1313774	SAMN50908454	Unknown_BE708-08M0136
SRR35221802	SRP616507	PRJNA1313774	SAMN50908455	Unknown_BE708-08M0137
SRR35221801	SRP616507	PRJNA1313774	SAMN50908456	Unknown_BE708-08M0138
SRR35221800	SRP616507	PRJNA1313774	SAMN50908457	Unknown_BE708-08M0139
SRR35221798	SRP616507	PRJNA1313774	SAMN50908458	Unknown_BE708-08M0140
SRR35221797	SRP616507	PRJNA1313774	SAMN50908459	Unknown_BE708-08M0141
SRR35221796	SRP616507	PRJNA1313774	SAMN50908460	Unknown_BE708-08M0142
SRR35221795	SRP616507	PRJNA1313774	SAMN50908461	Unknown_BE708-08M0143
SRR35221793	SRP616507	PRJNA1313774	SAMN50908462	Unknown_BE708-08M0144
SRR35221763	SRP616507	PRJNA1313774	SAMN50908463	Unknown_BE708-08M0145
SRR35221762	SRP616507	PRJNA1313774	SAMN50908464	Unknown_BE708-08M0146
SRR35221761	SRP616507	PRJNA1313774	SAMN50908465	Unknown_BE708-08M0147
SRR35221781	SRP616507	PRJNA1313774	SAMN50908466	Unknown_BE708-08M0148
SRR35221780	SRP616507	PRJNA1313774	SAMN50908467	Unknown_BE708-08M0149
SRR35221779	SRP616507	PRJNA1313774	SAMN50908468	Unknown_BE708-08M0150
SRR35221778	SRP616507	PRJNA1313774	SAMN50908469	Unknown_BE708-08M0151
SRR35221799	SRP616507	PRJNA1313774	SAMN50908470	Unknown_BE708-08M0152
SRR35221777	SRP616507	PRJNA1313774	SAMN50908471	Unknown_BE708-08M0153

SRR35221775	SRP616507	PRJNA1313774	SAMN50908472	Unknown_BE708-08M0154
SRR35221774	SRP616507	PRJNA1313774	SAMN50908473	Unknown_BE708-08M0155
SRR35221773	SRP616507	PRJNA1313774	SAMN50908474	Unknown_BE708-08M0156
SRR35221772	SRP616507	PRJNA1313774	SAMN50908475	Unknown_BE708-08M0157
SRR35221771	SRP616507	PRJNA1313774	SAMN50908476	Unknown_BE708-08M0158
SRR35221770	SRP616507	PRJNA1313774	SAMN50908477	Unknown_BE708-08M0159
SRR35221769	SRP616507	PRJNA1313774	SAMN50908478	Unknown_BE708-08M0160
SRR35221768	SRP616507	PRJNA1313774	SAMN50908479	Unknown_BE708-08M0161
SRR35221767	SRP616507	PRJNA1313774	SAMN50908480	Unknown_BE708-08M0162
SRR35221766	SRP616507	PRJNA1313774	SAMN50908481	Unknown_BE708-08M0163
SRR35221764	SRP616507	PRJNA1313774	SAMN50908482	Unknown_BE708-08M0164
SRR35221742	SRP616507	PRJNA1313774	SAMN50908483	Unknown_BE708-08M0165
SRR35221741	SRP616507	PRJNA1313774	SAMN50908484	Unknown_BE708-08M0166
SRR35221738	SRP616507	PRJNA1313774	SAMN50908485	Unknown_BE708-08M0167
SRR35221737	SRP616507	PRJNA1313774	SAMN50908486	Unknown_BE708-08M0168
SRR38918449	SRP705546	PRJNA1472955	SAMN60527697	SF250331DKZ046
SRR38918452	SRP705546	PRJNA1472955	SAMN60527694	SF250331DKZ043
SRR38918453	SRP705546	PRJNA1472955	SAMN60527693	SF250331DKZ042
SRR38918456	SRP705546	PRJNA1472955	SAMN60527691	SF250331DKZ040
SRR38918459	SRP705546	PRJNA1472955	SAMN60527688	SF250331DKZ037
SRR38918463	SRP705546	PRJNA1472955	SAMN60527684	SF250331DKZ033
SRR38918464	SRP705546	PRJNA1472955	SAMN60527683	SF250331DKZ032
SRR38918468	SRP705546	PRJNA1472955	SAMN60527680	SF250331DKZ029
SRR38918472	SRP705546	PRJNA1472955	SAMN60527676	SF250331DKZ025
SRR38918479	SRP705546	PRJNA1472955	SAMN60527670	SF240920DKZ019
SRR38918484	SRP705546	PRJNA1472955	SAMN60527665	SF240920DKZ014
SRR38918487	SRP705546	PRJNA1472955	SAMN60527662	SF240920DKZ011
SRR38918411	SRP705546	PRJNA1472955	SAMN60527660	SF240920DKZ009
SRR38918470	SRP705546	PRJNA1472955	SAMN60527678	SF250331DKZ027
SRR38918401	SRP705546	PRJNA1472955	SAMN60527741	SF240920DKZ046
SRR38918404	SRP705546	PRJNA1472955	SAMN60527738	SF240920DKZ043
SRR38918405	SRP705546	PRJNA1472955	SAMN60527737	SF240920DKZ042
SRR38918407	SRP705546	PRJNA1472955	SAMN60527735	SF240920DKZ040
SRR38918410	SRP705546	PRJNA1472955	SAMN60527732	SF240920DKZ037
SRR38918414	SRP705546	PRJNA1472955	SAMN60527729	SF240920DKZ033
SRR38918415	SRP705546	PRJNA1472955	SAMN60527728	SF240920DKZ032
SRR38918417	SRP705546	PRJNA1472955	SAMN60527726	SF240920DKZ030
SRR38918421	SRP705546	PRJNA1472955	SAMN60527722	SF240920DKZ025
SRR38918428	SRP705546	PRJNA1472955	SAMN60527716	SF250331DKZ019
SRR38918434	SRP705546	PRJNA1472955	SAMN60527711	SF250331DKZ014
SRR38918437	SRP705546	PRJNA1472955	SAMN60527708	SF250331DKZ011
SRR38918439	SRP705546	PRJNA1472955	SAMN60527706	SF250331DKZ009
SRR38918419	SRP705546	PRJNA1472955	SAMN60527724	SF240920DKZ027

Table S5. Relationships between environmental distance and |βNTI|.

Group	Biogeomorphy states	R^2	*p* value	Trend
3-16S	Poor	0.002	0.736	Negative
3-16S	Moderate	0.009	0.578	Negative
3-16S	Good	0.011	0.535	Negative
3-18S	Poor	0.147	0.004	Positive
3-18S	Moderate	0.003	0.756	Negative
3-18S	Good	0.020	0.414	Positive
022-16S	Poor	0.052	0.321	Positive
022-16S	Moderate	0.002	0.811	Positive
022-16S	Good	0.125	0.035	Negative
022-18S	Poor	0.105	0.152	Negative
022-18S	Moderate	0.109	0.049	Negative
022-18S	Good	0.005	0.691	Positive

R^2, *p* values, and trend directions were obtained from regressions of |βNTI| against pairwise environmental distance across microbial groups and reef biogeomorphy states.

Table S6. Sample sizes used in each analysis.

Group	Original sample size	Balanced sample size
NearCR	86	54
OffCR	28	28
Poor	50	18
Moderate	18	18
Good	18	18

Table S7. Detailed sample information and distance to the reef core zone.

SampleID	Group	Biogeomorphic status	Distance (Km)
Sample1	NearCR	Poor	0
Sample2	NearCR	Poor	0
Sample3	NearCR	Poor	0
Sample4	NearCR	Poor	0
Sample5	NearCR	Poor	0
Sample6	NearCR	Poor	0
Sample7	NearCR	Poor	0
Sample8	NearCR	Poor	0
Sample9	NearCR	Poor	0
Sample10	NearCR	Poor	0
Sample11	NearCR	Moderate	0
Sample12	NearCR	Moderate	0
Sample13	OffCR	Moderate	1.18
Sample14	OffCR	Moderate	1.18
Sample15	NearCR	Moderate	0.317
Sample16	NearCR	Moderate	0.317
Sample17	NearCR	Poor	0.39
Sample18	NearCR	Poor	0.39
Sample19	NearCR	Moderate	0.41
Sample20	NearCR	Moderate	0.41
Sample21	NearCR	Good	0.147
Sample22	NearCR	Good	0.147
Sample23	NearCR	Moderate	0
Sample24	NearCR	Moderate	0
Sample25	OffCR	Moderate	2.28
Sample26	OffCR	Moderate	2.28
Sample27	OffCR	Moderate	0.644
Sample28	OffCR	Moderate	0.644
Sample29	NearCR	Poor	0
Sample30	NearCR	Poor	0
Sample31	NearCR	Poor	0
Sample32	NearCR	Poor	0
Sample33	NearCR	Poor	0
Sample34	NearCR	Poor	0
Sample35	NearCR	Good	0
Sample36	NearCR	Good	0
Sample37	OffCR	Good	0.588
Sample38	OffCR	Good	0.588
Sample39	OffCR	Good	1.53
Sample40	OffCR	Good	1.53

Sample41	NearCR	Good	0
Sample42	NearCR	Good	0
Sample43	NearCR	Moderate	0.2
Sample44	NearCR	Moderate	0.2
Sample45	NearCR	Poor	0
Sample46	NearCR	Poor	0
Sample47	NearCR	Poor	0
Sample48	NearCR	Poor	0
Sample49	NearCR	Poor	0
Sample50	NearCR	Poor	0
Sample51	NearCR	Poor	0
Sample52	NearCR	Poor	0
Sample53	NearCR	Poor	0
Sample54	NearCR	Poor	0
Sample55	NearCR	Moderate	0
Sample56	NearCR	Moderate	0
Sample57	OffCR	Poor	0.687
Sample58	OffCR	Poor	0.687
Sample59	NearCR	Poor	0
Sample60	NearCR	Poor	0
Sample61	OffCR	Ocean	211.92
Sample62	OffCR	Ocean	211.92
Sample63	OffCR	Ocean	43.58
Sample64	OffCR	Ocean	43.58
Sample65	OffCR	Poor	0.717
Sample66	OffCR	Poor	0.717
Sample67	NearCR	Moderate	0
Sample68	NearCR	Moderate	0
Sample69	NearCR	Poor	0
Sample70	NearCR	Poor	0
Sample71	OffCR	Poor	1.02
Sample72	OffCR	Poor	1.02
Sample73	OffCR	Poor	0.751
Sample74	OffCR	Poor	0.751
Sample75	NearCR	Poor	0
Sample76	NearCR	Poor	0
Sample77	NearCR	Poor	0.438
Sample78	NearCR	Poor	0.438
Sample79	NearCR	Poor	0
Sample80	NearCR	Poor	0
Sample81	NearCR	Poor	0.35
Sample82	NearCR	Poor	0.35
Sample83	NearCR	Good	0

Sample84	NearCR	Good	0
Sample85	NearCR	Good	0
Sample86	NearCR	Good	0
Sample87	NearCR	Good	0
Sample88	NearCR	Good	0
Sample89	NearCR	Good	0.322
Sample90	NearCR	Good	0.322
Sample91	NearCR	Good	0.242
Sample92	NearCR	Good	0.242
Sample93	NearCR	Good	0
Sample94	NearCR	Good	0
Sample95	NearCR	Moderate	0
Sample96	NearCR	Moderate	0
Sample97	NearCR	Poor	0
Sample98	NearCR	Poor	0
Sample99	NearCR	Poor	0
Sample100	NearCR	Poor	0
Sample101	OffCR	Ocean	164.6
Sample102	OffCR	Ocean	164.6
Sample103	NearCR	Poor	0
Sample104	NearCR	Poor	0
Sample105	NearCR	Poor	0
Sample106	NearCR	Poor	0
Sample107	NearCR	Poor	0
Sample108	NearCR	Poor	0
Sample109	NearCR	Moderate	0
Sample110	NearCR	Moderate	0
Sample111	OffCR	Ocean	92.2
Sample112	OffCR	Ocean	92.2
Sample113	OffCR	Ocean	16.91
Sample114	OffCR	Ocean	16.91

Table S8. Biomarker ASVs retained in state-specific co-occurrence networks.

Biogeomorphic status	Total biomarker ASVs	Peripherals	Module hubs	Network hubs
Poor	22	7	0	0
Moderate	40	20	0	0
Good	42	22	0	0
